# Topology‐Optimized Stretchable Piezoelectric Sensors With Tailored Liquid‐Metal Circuits for Anisotropic Stress‐Adaptive Motion Monitoring

**DOI:** 10.1002/adma.202518168

**Published:** 2026-02-07

**Authors:** Hanmin Zeng, Qianqian Xu, Jianxun Zhang, Peiqiong Zhou, Jiachen Zhang, Jinlan Li, Senfeng Zhao, Kechao Zhou, Dou Zhang, Chris Bowen, Yan Zhang

**Affiliations:** ^1^ State Key Laboratory of Powder Metallurgy Central South University Changsha Hunan China; ^2^ Hunan Provincial Key Laboratory of Micro & Nano Materials Interface Science College of Chemistry and Chemical Engineering Central South University Changsha Hunan China; ^3^ Department of Mechanical Engineering University of Bath Bath UK

**Keywords:** liquid‐metal printing, motion monitoring, self‐powered sensor, stretchable piezoelectric sensors

## Abstract

The design of high‐sensitivity stretchable piezoelectric sensors remains challenging due to the inherent trade‐off between the ability to achieve high levels of mechanical deformation while maintaining efficient stress transduction. Here, we propose a new topology‐optimization strategy to construct stretchable piezoelectric sensors that efficiently utilize the spatial stress distribution and are able to adapt to a range of anisotropic mechanical stress states. By exploiting computer‐aided topology optimization, the distribution of piezoelectric ceramic units within the sensor was tailored to maximize the degree of stress transfer, resulting in an increase of 103.5% and 59.7% in the maximum piezoelectric potential when subject to tension and torsion, respectively. To ensure structural stretchability and adaptability of the topology optimized sensors when subject to complex loading environments, a direct ink writing process was developed to create stretchable eutectic gallium‐indium liquid alloy (EGaIn) electrodes. Based on a shear‐driven mechanism of printing, new predictive theoretical equations governing printing performance were developed that could predict the printed state (with 94.7% accuracy) and enable trace width control (relative error < 15%). The final optimized sensor exhibited excellent sensitivity, achieving 14.0 V per strain and 0.10 V per degree when subject to tensile and torsional loads, exceeding the unoptimized device by 59.2% and 92.4%, respectively. Finally, inspired by the morphological characteristics of butterflies and guided by the topology‐optimized layout, a multi‐channel sensor was constructed to accurately identify the pattern and amplitude of a complex range of neck movements, demonstrating the significant potential of the new design and manufacturing approach for wearable electronics.

## Graphical abstract

Schematic showing the leveraging of topology optimization, the printing of a tailored eutectic gallium‐indium liquid alloy electrode, and porous ceramic engineering to simultaneously enhance mechanical durability, stretchability, and sensing sensitivity to produce a highly deformable and stretchable sensor capable of monitoring complex and anisotropic deformation. This new sensor architecture was successfully employed for the precise identification of the type of neck motion, and its amplitude.

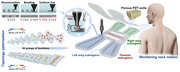

## 1 Introduction

Piezoelectric sensors have emerged as a technological frontier in next‐generation wearable electronics [1, 2, 3, 4], due to their unique electromechanical conversion capabilities [5, 6, 7] and reliable signal acquisition [8, 9]. While significant progress has been made in the development of piezoelectric sensors, the inherent rigidity of conventional piezoelectric sensors [10] hinders their application in complex loading environments. In contrast, stretchable piezoelectric sensors exhibit a high level of mechanical flexibility, a wide measurement range [11], high durability [12], and strong environmental adaptability [13]. These attributes enable their widespread application in areas such as medical monitoring [14], biomechanical capture, wearables [11, 12, 13], and sensor networks for the Internet of Things (IoT) [13, 15, 16, 17].

For the construction of stretchable piezoelectric sensors, strategies employed to date include incorporating piezoelectric micro‐ or nano‐materials into elastic polymers [18], or fabricating flexible piezoelectric polymers into pre‐stretched structures [13, 19, 20, 21]. The open‐circuit voltage (*V*_oc_) and short‐circuit current (*I*_sc_) of piezoelectric sensors are positively correlated with mechanical stress [22, 23]:

(1) $$V_{\mathrm{oc}}=\frac{d_{33}}{\varepsilon_{33}^{T}}\;\times d\times \Delta \sigma$$

(2) $$I_{\mathrm{sc}}=d_{33}\;\times A\times \frac{\Delta \sigma }{\Delta t}$$

where *d*_33_ is the piezoelectric coefficient; $\varepsilon_{33}^{T}$ is the relative permittivity at constant stress, *d* is the effective thickness of the piezoelectric material along the electric field direction, *A* is the effective electrode area, Δ*σ* is the stress change and Δ*t* is the corresponding time interval. As a result, the introduction of an inert and inactive polymer, that reduces the piezoelectric coefficient, or a reduction of local stress or strain results in a decrease in the electrical signal [24, 25]. In rigid devices, which typically respond to compressive stimuli, a uniform distribution of piezoelectric units is essential to maintain a stable and reproducible sensor response [26, 27]. However, the high deformability of a stretchable piezoelectric sensors enables a broad range of mechanical responses to be monitored [28], including tension, bending, and torsion [12]. Under such distributed loading conditions, a uniform arrangement of the piezoelectric units often results in large regions being subjected to minimal or negligible strain levels [29, 30], which can be described as “dead zones” [31] as they do not lead to a sensor response. To address this limitation, the integration of optimization methods with computational modeling is necessary to maximize the efficiency of the utilization of a distributed load across the piezoelectric elements, thereby enhancing the overall sensing performance of any stretchable piezoelectric system [32, 33].

Topological analysis provides a deep descriptive tool to understand the connections and relationships between the spatial distribution of structural units in a sensor system [34] and its influence on the overall properties [35]; this is particularly important in optimizing the utilization of internal spatial stress [36, 37]. For example, Hu et al. [38] proposed a gyro‐shaped lattice support structure based on topological optimization, which reduced the deformation of the beam tip by 69% and 58% compared with the uniform lattice support and the all‐solid support, respectively. Recent studies have shown that topological optimization has the potential to improve the effective piezoelectric coefficient. As an example, He et al. [39] performed topology optimization on the distribution of piezoelectric material in the thickness direction of a plate‐type piezoelectric energy harvester that was subject to harmonic excitation, and theoretical results showed that the energy conversion efficiency was improved by approximately 800%.

While topology optimization offers a promising pathway to maximize the capture and utilization of a distributed load, it imposes stringent requirements on the structural distribution of a stretchable piezoelectric sensor, in particular with regard to the electrode structure, which needs to meet the dual requirements of providing both mechanical stretchability and design adaptability [40].

In this context, the eutectic gallium indium (EGaIn) alloy is a liquid metal that has both metallic and fluid properties [41]. Compared with conventional conductive materials such as gold, copper, carbon nanotubes, and graphene, the EGaIn alloy offers the unique combination of high electrical conductivity and exceptional mechanical stretchability, making it an attractive candidate for a stretchable electrode in sensor applications [42]. In addition, as a result of the fluidic nature of the EGaIn alloy, the resulting mechanical constraints are negligible [41, 43]. However, the precise patterning and fabrication of the EGaIn electrode faces a number of challenges due to the high surface tension and the spontaneous formation of a surface oxide layer [41].

**FIGURE 1:**
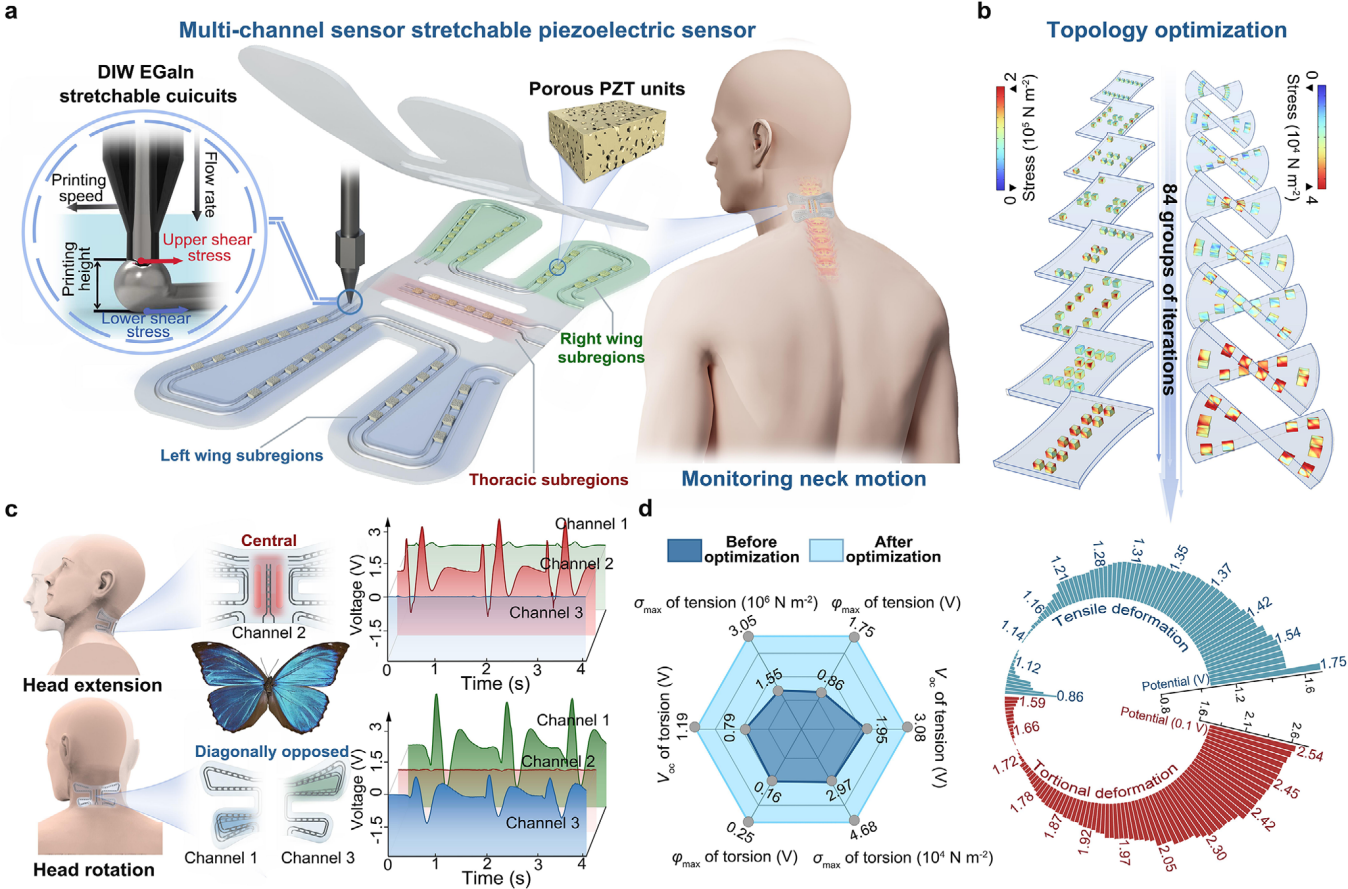
Design and performance of a multi‐channel stretchable piezoelectric sensor. a) Schematic of topology‐optimized stretchable piezoelectric sensor. b) Iterative evolution of stress distribution during topology optimization when subject to tensile and torsional deformation modes of the sensors. c) multi‐channel voltage signals are collected by the butterfly‐morphology‐inspired sensor during head extension and left rotation. Channels 1, 2, and 3 collect signals from the left, central, and right subregions, respectively. d) Compared with the sensor before optimization within the same tensile and torsional deformation, the optimized sensor exhibits excellent performance in terms of maximum stress (*σ*_max_), maximum potential (*φ*_max_) and open‐circuit voltage (*V*_oc_). e) *φ*_max_ of 84 groups of iteration under tensile and torsional deformation.

Herein, we propose a topology optimization strategy for fabricating stretchable piezoelectric sensors with an optimized distribution of material that is tailored to provide anisotropic mechanical adaptability, thereby enabling efficient capture of mechanical signals. By integrating computer‐aided topology optimization with finite element analysis (FEA), we investigated in detail the mechanical response of the piezoelectric component when subjected to distributed loads. Automated simulations comprising 84 groups of iterative analyses per deformation mode yielded an optimized assembly and design of the sensor that increased the maximum stress (*σ*_max_) and maximum piezoelectric potential (*φ*_max_) on the piezoelectric unit by 96.8% and 103.5%, respectively. In contrast to conventional stress analysis, this topology optimization process enables overall optimization of soft‐rigid hybrid assembly layout in stretchable sensors, thereby establishing optimal stress transfer pathways for targeted deformation modes. As a model system, we selected a composite structure consisting of a porous lead zirconate titanate (PZT) ceramic embedded in a mechanically flexible Ecoflex elastomer, where the method is readily extended to a range of materials and structural configurations. To meet the stringent design freedom that is required by topology optimization for stretchable electrodes, we systematically analyzed the dynamic behavior of the EGaIn electrode printing process. Based on a shear‐driven mechanism of printing, we derived predictive theoretical equations governing printing performance, including the printed state (with 94.7% accuracy) and trace width control (relative error < 15%). The printed EGaIn electrodes exhibited only a 0.11% change in resistance after over 22,000 tensile cycles at 60% strain. The resulting stretchable piezoelectric sensor integrates a topologically optimized assembly layout with precision‐patterned EGaIn electrodes and achieves 14.0 V per strain and 0.10 V per degree, demonstrating 59.2% and 92.4% higher sensitivity when subjected to tensile and torsional deformation, respectively, compared to unoptimized devices. Finally, inspired by the bilaterally symmetrical and functionally partitioned butterfly wings, the sensor was divided into multiple subregions with region‐specific topology‐optimized layouts, which enhance the degree of localized stress coupling. As a wearable sensor, the central subregion of the sensor is able to responds primarily to stretching of a human neck, while the two lateral sub‐regions are able to capture the left and right torsional motion of the neck. By integrating the waveform features and amplitudes obtained from multi‐channel signal acquisition, this bioinspired architecture enables precise identification of neck motion types and magnitudes, highlighting the applicability of our new approach in the design and fabrication of stretchable sensors for future wearable electronics (Figure 1).

## 2 Results and Discussion

### 2.1 Topology‐Optimized Layout for Anisotropic Mechanical Responses

Computer‐aided topology optimization was employed to construct the design space of the PZT‐Ecoflex composites under specific constraints, as detailed below, and perform further iterative solutions in finite element analysis (FEA) to determine the optimal assembly layout. Specifically, PZT cubes were used as the piezoelectric component and fully encapsulated within the flexible Ecoflex matrix, which defined the design space for the optimization process. For the two typical anisotropic mechanical deformation modes, namely tension and torsion, 12 PZT piezoelectric units were arranged in the design space. In addition, the assembly layout of the piezoelectric units was designed according to three principles. First, to ensure a symmetric stress distribution and avoid excessive concentration of units in the sensor, the units were place symmetrically along both central axes of the design space. Second, to prevent overlap or excessively small spacing during simulation, the minimum distance between adjacent units was set equal to the edge length of single unit. Third, to maintain the effectiveness of topology optimization, the design space was defined as a square with a side length equal to the number of units multiplied by the size of each unit. Finally, the maximum stress (*σ*_max_) and maximum potential (*φ*_max_) on the piezoelectric component when subjected to the same strain were used as iterative targets to obtain the optimal utilization layout of the distributed load. Under the above constraints, the optimal assembly layout was obtained after evolution of 84 groups of iterations (Movie S1).

**FIGURE 2:**
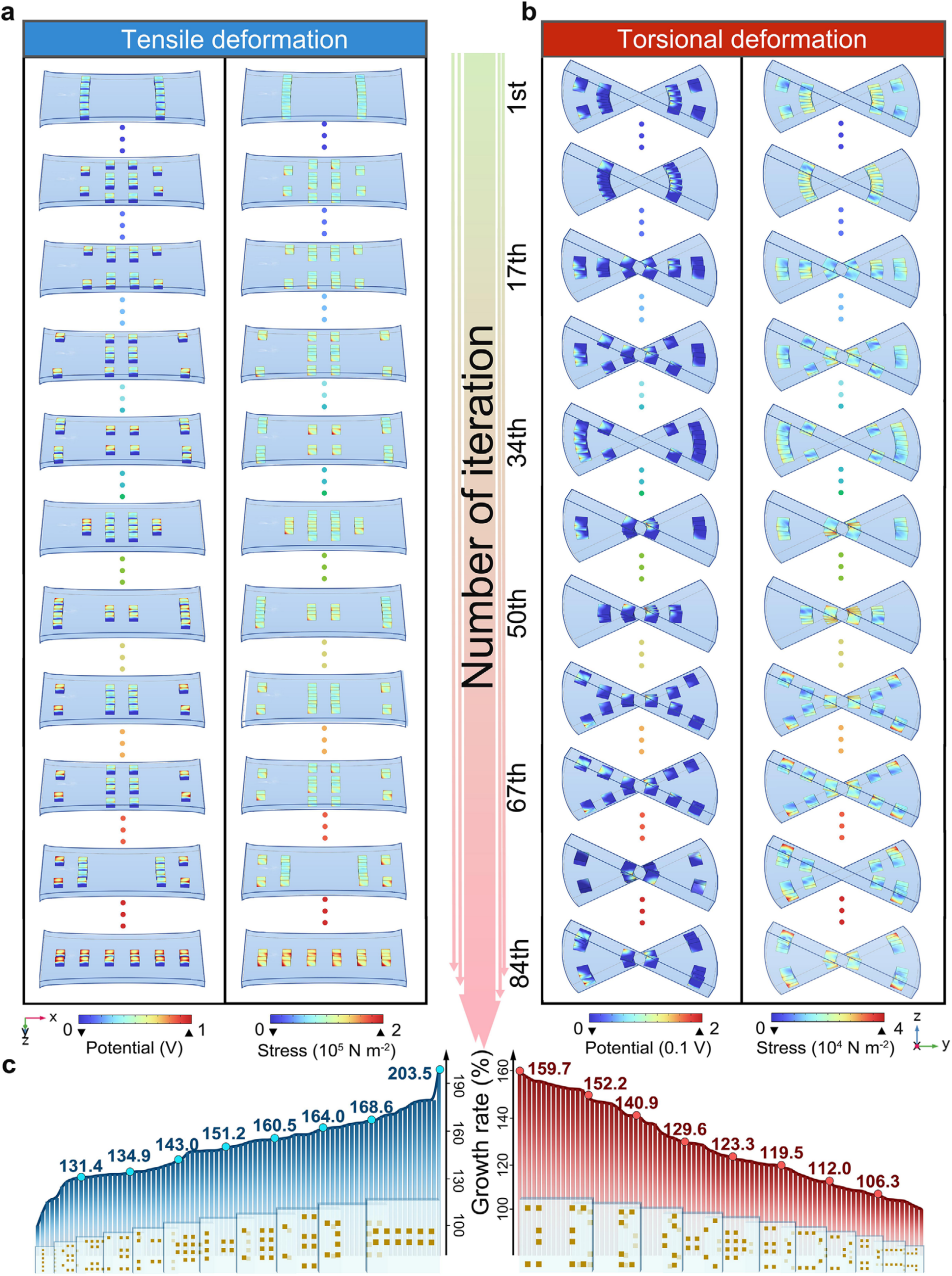
Finite element analysis (FEA) results as a result of topology optimization iterations when subjected to tensile and torsional deformation. FEA results of stress and piezoelectric potential distribution obtained for the 1st, 8th, 17th, 25th, 34th, 42nd, 50th, 59th, 67th, 76^th^, and 84th iterations subjected to a) tensile deformation and b) torsional deformation. c) Evolution of *φ*_max_ among the 84 groups of iteration subjected to tensile deformation (left) and torsional deformation (right).

The assembly layout obtained from the 1st, 8th, 17th, 25th, 34th, 42nd, 50th, 59th, 67th, 76th and 84th iterations and the corresponding FEA results are shown in Figure 2a,b. The complete iteration results are provided in Figures S1–S4. The results reveal a progressive increase in the *σ*_max_ on the piezoelectric component, accompanied by a corresponding rise in *φ*_max_ with increasing *σ*_max_. As shown in Figure 2c, when the sensor is subjected to tensile deformation, the piezoelectric array with the optimal assembly layout exhibits a significantly larger *σ*_max_ and *φ*_max_, which increased by 96.8% and 103.5%, respectively. The developed topology optimization strategy demonstrates a significant improvement in stress utilization during torsion. The *σ*_max_ increased from 2.97 × 10^4^ N m^−2^ to 4.68 × 10^4^ N m^−2^, and the *φ*_max_ increased from 0.16 V to 0.25 V, which represents an increase of 57.6% and 59.7%, respectively. In addition, ceramic units based on different shapes consistently converge to the same optimal arrangement (Figures S5, S6), thereby indicating that the geometry of individual PZT units has a negligible impact to the overall topology optimization. Since cubes are the most common ceramic shape, and most easy to fabricate, we selected a cube geometry for design and optimization in subsequent experiments. By comparing the optimization results of the PZT‐Ecoflex composites with the *σ*_max_ of the Ecoflex elastomer (Figures S7,S8), it can be observed that the distribution of location of the piezoelectric units in the composites is not consistent with the stress concentration areas in the elastomer. This phenomenon is attributed to the elastic modulus of the soft‐rigid hybrid material differing from a homogeneous material [10, 31, 44]. The strain distribution in the piezoelectric units is also closely related to the corresponding stress field (Figure S9). When compared to a fully flexible or fully rigid sensor, the hybrid soft‐rigid sensors combine components with different Young's moduli [43], where the spatial arrangement of the rigid piezoelectric units is able to alter the overall/effective modulus of the sensor when subjected to anisotropic deformation. These results demonstrate that regulating the assembly and relationship between the two materials of contrasting elastic moduli (*E*_PZT_/*E*_Ecoflex_ ≈ 10^5^) through a topological optimization strategy can effectively improve the stress transfer path and utilization, thereby concentrating stress efficiently on the piezoelectric unit and potentially achieving significant enhancements in piezoelectric output, sensitivity and adaptability.

### 2.2 Precisely Tailored Printing of Stretchable EGaIn Electrode Circuits

**FIGURE 3:**
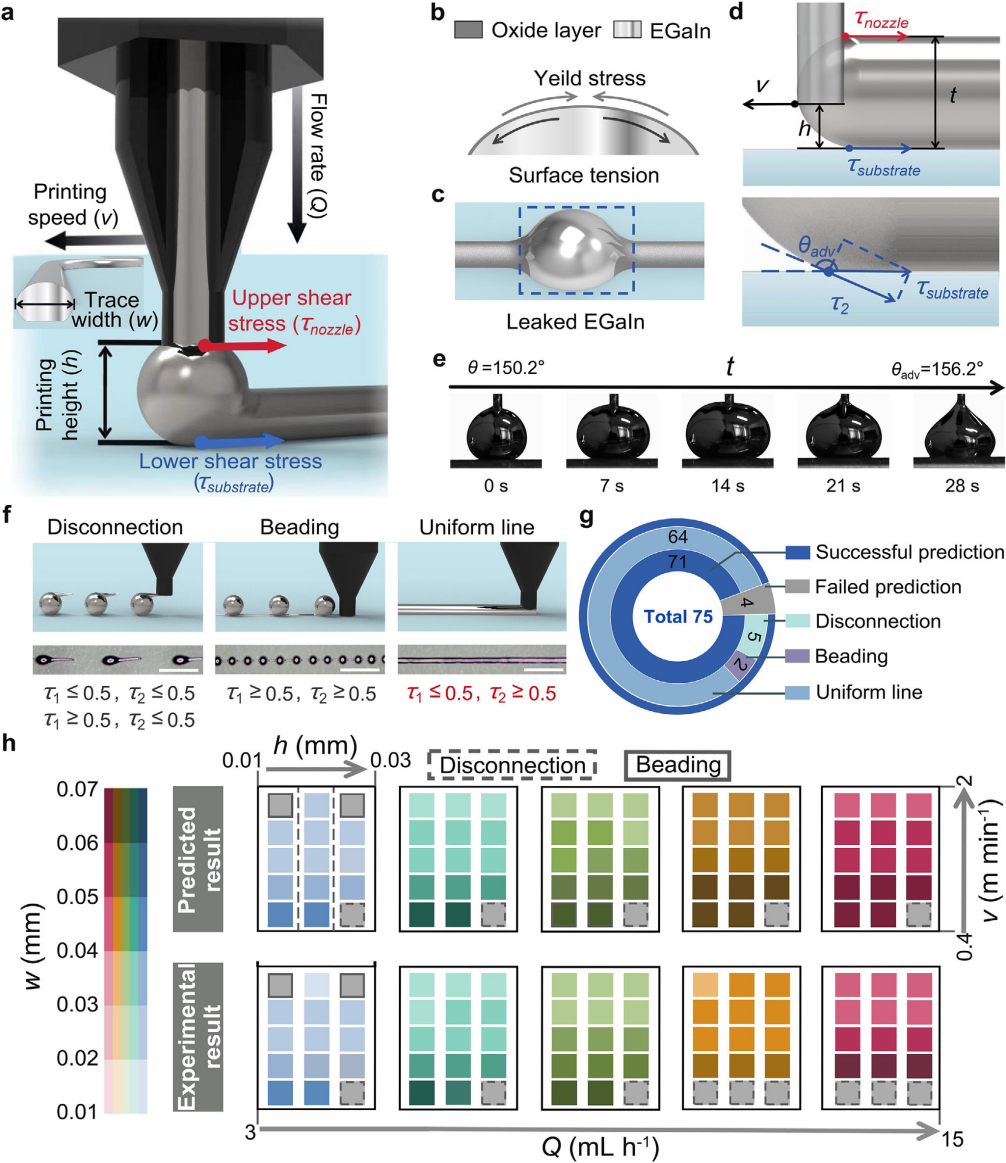
Mechanism analysis and parameter optimization for the printing process to produce precisely tailored eutectic gallium indium (EGaIn) electrode circuits. a) Schematic of EGaIn 5printing subjected to the synergistic effects of multiple parameters and stress. b) EGaIn exhibits high surface tension, and the native oxide layer provides sufficient mechanical strength to maintain the stability. c) EGaIn rapidly leaks, while the oxide layer is ruptured. d) Force analysis of the shear stress acting on the upper surface (top) and lower surfaces (bottom) of EGaIn during printing of circuits. e) advancing contact angle (*θ*_adv_) and receding contact angle (*θ*_rec_) of EGaIn on Ecoflex substrate measured by the sessile drop expansion‐contraction method. f) Schematic and optical image of the three typical printing states, including disconnection (left), beading (middle) and uniform line (right). Scale bars, 200 µm. g) Pie charts of printing prediction and success rate statistics. h) Relationship between the printing state and the printing parameters, prediction and experimental results are presented using a color block diagram.

To produce the optimized sensors, a direct int writing (DIW) technology was employed for the rapid and precisely tailored fabrication of stretchable electrodes [40]. As shown in Figure 3a, considering that the preparation of EGaIn electrode patterns is affected by the synergistic effects associated with multiple parameters, including extrusion speed, printing speed, and printing height. To illustrate the printing process, Movie S2 is provided, and accordingly, a detailed analysis of the dynamics of EGaIn extrusion was performed. Once the EGaIn liquid alloy is exposed to air, a dense oxide layer (1–5 nm) rapidly forms on its surface, which forms a thin skin. This oxide layer supports the internal liquid, providing a solid‐like morphology and stability, where it exhibits a yield stress (*σ*) of approximately 0.5 N m^−1^ [41] (Figure 3b). When the EGaIn is extruded from the nozzle tip during printing, it experiences a shear stress generated by the relative motion of the EGaIn and the nozzle (*τ*_nozzle_) as well as between the EGaIn and the substrate (*τ*_substrate_); see Figure 3a. When the tangential component of the surface stress (tangential component of *τ*_nozzle_ is defined as *τ*_1_, and tangential component of *τ*_substrate_ is defined as *τ*_2_) exceeds the yield stress, the oxide layer will rupture. For the upper surface of the EGaIn, once the oxide layer is ruptured, the EGaIn will rapidly leak from the printed circuit, driven by its inherent high surface tension (Figure 3c). For the lower surface of the EGaIn, a sufficient level of stress is required to overcome the constraint of the oxide layer, driving the EGaIn so that it can extrude continuously and uniformly. The re‐formation of the oxide layer on the substrate contributes to maintaining the stability of the electrode circuit that is being printed. Therefore, the key to achieving precisely tailored printing of EGaIn electrode circuits lies in the careful regulation of the state of stress (*τ*_1_, *τ*_2_ and *σ*). As shown in Figure 3d, when the EGaIn is extruded from the nozzle, the upper surface shear stress, *τ*_1_, can be defined as:

(3) $$\tau_{1}=\eta \frac{v}{\sqrt{\frac{4kQ}{0.97\pi v}}-h}$$

where *v* is the printing speed, *Q* is the flow rate. *η* is the viscosity of EGaIn, *k* is the aspect ratio of the printed trace and *h* is the printing height. When the EGaIn contacts the substrate, the lower shear stress at the substrate, *τ*_2_, is defined as:

(4) $$\tau_{2}=\eta \frac{\cos\;\left(\pi -\theta_{\mathrm{adv}}\right)}{h}v$$

where *θ*_adv_ is the advancing contact angle of EGaIn on Ecoflex substrate (Figure 3e), additional details on the approach employed to determine the shear stress are outlined in the Experimental section.

According to Equations (3) and (4), a critical balance between *τ* and *σ* can be achieved by optimizing *Q*, *h*, and *v*. As shown in Figure 3f, the printed electrode circuits will theoretically exhibit the following three states: (i) EGaIn disconnection. When *τ*_2_ ≤ 0.5 (when *τ*_1_ ≤ 0.5 or *τ*_1_ ≥ 0.5), a continuous flow of the EGaIn cannot be maintained, and the printed line becomes discontinuous, thereby leading to the formation of separated EGaIn droplets (Figure S12); (ii) EGaIn beading. When *τ*_1_ ≥ 0.5 and *τ*_2_ ≥ 0.5, the EGaIn flows continuously from the nozzle; however, rupture of the oxide layer on the upper surface will lead to EGaIn leakage, thereby producing a thin printed line with EGaIn droplets attached (Figure S13); (iii) Formation of continuous and uniform EGaIn lines. When *τ*_1_ ≤ 0.5 and *τ*_2_ ≥ 0.5, the EGaIn flows continuously while the oxide layer on the upper surface remains intact, thereby enabling the printing of uniform and continuous EGaIn electrode lines. To investigate the accuracy of the proposed formula in predicting the printing states, theoretical analysis and corresponding experimental observations were conducted across a range of printing parameter combinations of *h*, *Q*, and *v*, thereby obtaining 75 sets of predictions and experimental results (Figure 3g,h). The color of each block indicates the printed trace width (*w*), while the border style denotes the printing status: a darker color corresponds to wider lines, no border indicates successful printing, a dotted border indicates interrupted printing, and a solid border indicates beaded printing (Figure 3h). As shown in Figure 3g, among the 75 comparisons between model and experimental observations, the observed printing states of 71 groups were consistent with the theoretical predictions, with a prediction accuracy of 94.7%. These results demonstrate that the above dynamic analysis can accurately predict the parameter range for the successful printing of EGaIn electrode circuits.

After obtaining the encouraging result outlined above, we further explored the reliability of the prediction of trace width, *w*, of the printed EGaIn electrode circuits. *w* can be quantified by Equation (5):

(5) $$w=\sqrt{\frac{4Q}{0.97k\pi v}}\;$$

**FIGURE 4:**
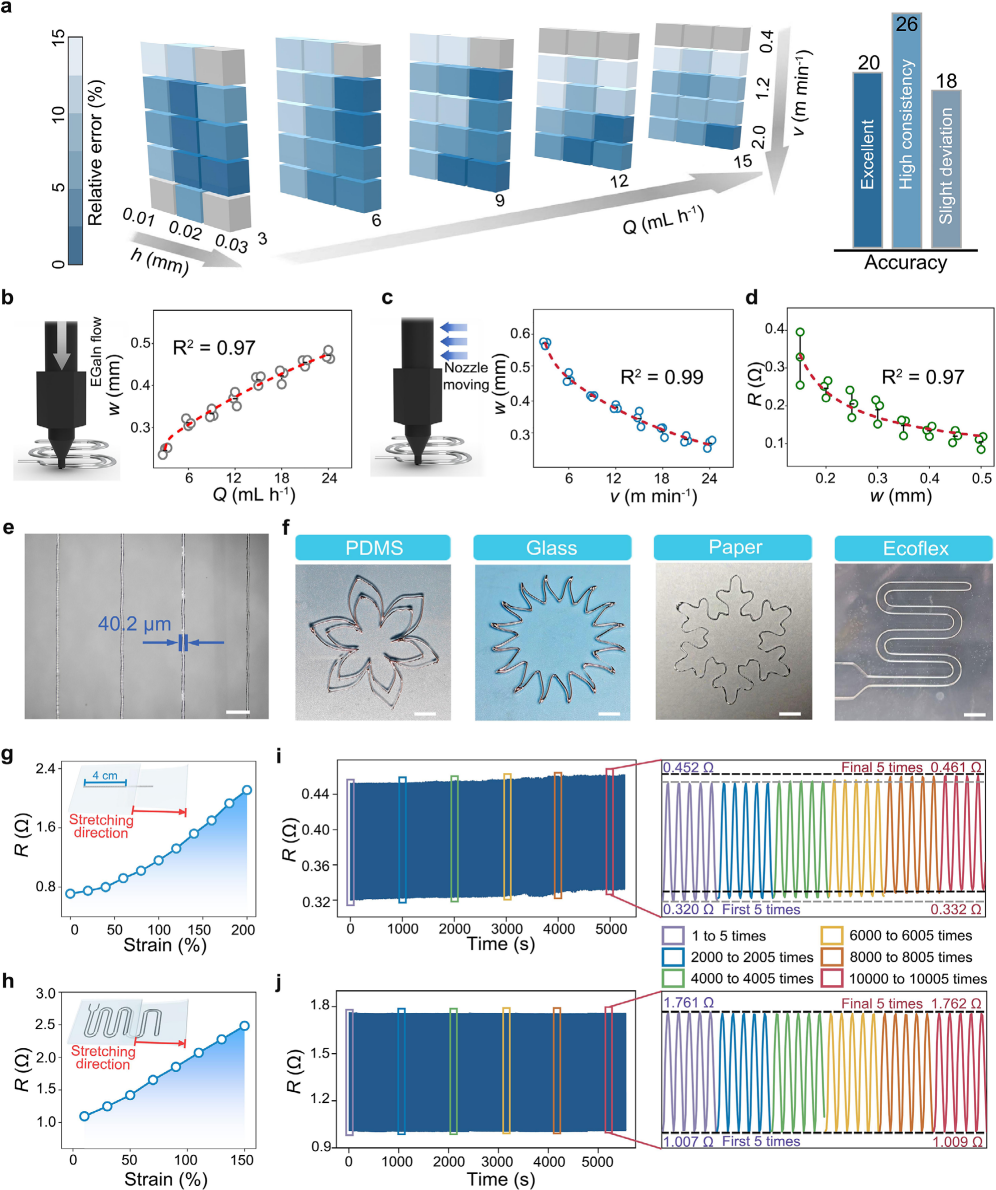
Electrical and mechanical properties of EGaIn electrode circuits. a) Relative error of the trace width *w*, between prediction and experimental results (left). Bar charts of prediction accuracy statistics. (right). Evaluation of the effects of parameters on the properties of printed lines, and the red curve represents a nonlinear fit to the averaged results of three repeated experiments: b) relationship between *Q* and *w* under a *v* of 2 m min^−1^ and a *h* of 0.02 mm. c) relationship between *v* and *w* under a *Q* of 6 mL h^−1^ and a printing *h* of 0.02 mm. d) Variation of line resistance with trace width, *w*, where the length of the measured lines are 4 cm. e) Printed lines with the thinnest trace width. Scale bar, 300 µm. f) Customized EGaIn for pattern printing, including the floral pattern on PDMS, solar pattern on glass, snowflake‐shaped pattern on paper, serpentine circuit on Ecoflex. Scale, 2 mm. Resistance of g) a 0.3 mm *w*, 4 cm long EGaIn straight line subjected to a maximum strain of 200% and h) serpentine circuit subjected to a maximum strain of 150%. Resistance changes of i) 2 cm EGaIn straight line and j) serpentine circuit after 22,000 cycles of stretching at 100% strain. Insets show details of resistance increase throughout the cycles, which determines the electrical and mechanical stability of the printed circuit.

Based on the established equation, the predicted trace widths can be readily calculated. Subsequently, experimental trace widths of corresponding printed samples were measured (Figures S14–S18, Support Information), and the relative deviations were calculated (Table S5). As shown in Figure 4a, among the 64 comparisons, 20 groups exhibited excellent accuracy with a relative error (*ε*) ≤ 5%, and 26 groups exhibited high consistency with *ε* ≤ 10%. Only 18 groups deviated slightly (*ε* ≤ 15%), which could be due to elevated *h* [45]. These findings strongly demonstrate that our prediction system not only guides the coordinated optimization of EGaIn printing parameters, but also provides quantitative guidance for printing a range of trace widths, *w*. To further elucidate the printing parameter‐trace width relationships, we performed a correlation analysis between the key printing variables and the measured *w*. As illustrated in Figure 4b, the *w* increases nonlinearly with increasing *Q* (R^2^ = 0.97), indicating that a lower EGaIn flow rate favors a higher printing resolution. In contrast, the *w* decreases nonlinearly with increasing printing speed *v* (R^2^ = 0.99); see Figure 4c.

Given this ability to finely control the printed *w*, we further evaluated the electrical characteristics of the circuit, namely the electrical conductivity and stability of the electrical properties with repeated strain. The resistance (*R*) of printed circuits can be quantified by Equation (6):

(6) $$R=\frac{8\rho L}{0.97k\pi w^{2}}\;$$

where *ρ* is the electrical conductivity, and *L* is the line length. According to Equation (6), the resistance is inversely proportional to the square of *w*. The experimental results in Figure 4d also show a similar trend, with the resistance decreasing nonlinearly with increasing *w* (R^2^ = 0.97), confirming the accuracy of the theoretical prediction. However, while the thinnest possible printable *w*, 40.2 µm, for the Ecoflex substrates (Figure 4e), the resistance fluctuations of the EGaIn circuits increase significantly as the *w* decreases (Figure S19). Therefore, 0.3 mm was selected as the optimal parameter for *w* to balance a good electrical stability and high circuit resolution. Guided by this predictive model, complex high‐precision patterns can be printed on various substrates with the optimized printing parameters. The advancing contact angle, *θ*_adv_, of EGaIn on polydimethylsiloxane (PDMS), glass, and paper were measured (Table S6), and the corresponding *τ_2_* were calculated based on Equation (5) (Tables S7–S8). Although EGaIn exhibits different wettability on these surfaces, the relationship between the calculated *τ_2_* and the *σ* remains unchanged, indicating that the printing parameter ranges established for Ecoflex are also applicable to other substrates. As shown in Figure 4f, this includes a floral pattern on polydimethylsiloxane (PDMS) film, a solar pattern on glass, and a snowflake‐shaped pattern on paper. We also printed a serpentine circuit for the preparation of actual sensors with multiple corners on the Ecoflex substrate.

Next, we further evaluated the dynamic stability of the precisely tailored circuit when subjected to mechanical deformation. As shown in Figure 4g, the electrical resistance of the straight EGaIn circuit (≈ 2 cm in length) increases by only 0.5 Ω when the tensile strain nonlinearly rises from 0% to 200%, demonstrating excellent tensile stability. According to Pouillet's law, the resistance depends on both the conductor length and its cross‐sectional area [46]. During stretching, the conductive path becomes longer while its cross‐section decreases due to the Poisson effect, leading to a slight increasing resistance. This explains the nonlinear trend observed in Figure 4g. In addition, we also measured serpentine circuits to better understand the resistance changes of EGaIn circuits with complex patterns when subjected to mechanical deformation. The resistance of the serpentine circuit linearly increased by approximately 1.5 Ω during application of a tensile strain from 0% to 150%, demonstrating that the EGaIn circuit continues to maintain a higher electrical stability when subject to a high level of strain (Figure 4i). In addition, both circuits exhibited high cycling stability, with resistance increases of only 5.70% and 0.71%, respectively, after more than 22,000 cycles at 100% strain (Figure 4h,j). Compared with previously reported work, the precise EGaIn circuits maintains excellent electrical stability when subject to high strain and cycling (Table S10).

### 2.3 Mechanisms Analysis and Electrical Properties

To fabricate the stretchable piezoelectric sensor, EGaIn conductive circuits were printed onto a low elastic modulus Ecoflex substrate. The electrodes of the pre‐polarized porous ceramic units were brought into direct contact with the printed circuits to establish a good electrical connectivity. After confirming stable electrical contact, the sensor was encapsulated with a layer of Ecoflex. To further enhance the sensor sensitivity, the introduction of pores into the dense piezoelectric ceramics is a low‐cost and up‐scalable approach to reduce the permittivity and thereby increase the piezoelectric voltage coefficient (*g*_33_) [27]. Porous PZT ceramics were prepared by the burnt‐out polymer spheres method. SEM and EDS images (Figures S20 and S21) exhibit a microstructure consisting of PZT ceramics with randomly, uniformly distributed spherical pores. The size and connectivity of the pores increased with the porosity volume fraction ranging from 40.9% to 60.7% [47]. Due to the introduction of low dielectric constant pores (air), the relative permittivity ($\varepsilon_{33}^{T}$) of all low‐porosity PZT ceramics decreases more than 15% with increasing porosity [48] (Figure S22). As shown in Figure S23, the dielectric loss of the porous PZT ceramics was less than 0.03, which ensured that the porous PZT ceramics maintain good dielectric properties for poling and sensing. Figure S24 shows the polarization‐electric field (*P*‐*E*) loops of the porous PZT ceramics, and all porosity levels exhibit excellent ferroelectricity. As the porosity increased from 40.9 vol% to 60.7 vol%, the remanent polarization (*P*_r_) of the porous PZT ceramics decreased from 10.2 µC cm^−2^ to 2.6 µC cm^−2^ and the maximum polarization (*P*_max_) decreased from 11.6 µC cm^−2^ to 3.2 µC cm^−2^ [49]. Figure S25 shows the variation of the piezoelectric coefficient (*d*_33_) and the piezoelectric voltage coefficient (*g*_33_) with porosity. The *d*_33_ decreased 24.9% from 567 pC N^−1^ to 426 pC N^−1^ with increasing pore volume fraction [50]. Conversely, the *g*_33_ increased by 113.1% from 84.6 mV m N^−1^ to 180.3 mV m N^−1^. The piezoelectric ceramic with a higher *g*_33_ can produce a higher open‐circuit voltage output when subjected to the same stress load [51]. However, limited by the inherent brittleness of ceramics, an excessive level of porosity will lead to structural failure under an applied stress [52]. Therefore, porous ceramics with porosity of 50 vol% represent an optimum trade‐off between sensing sensitivity and mechanical stability for sensor applications.

**FIGURE 5:**
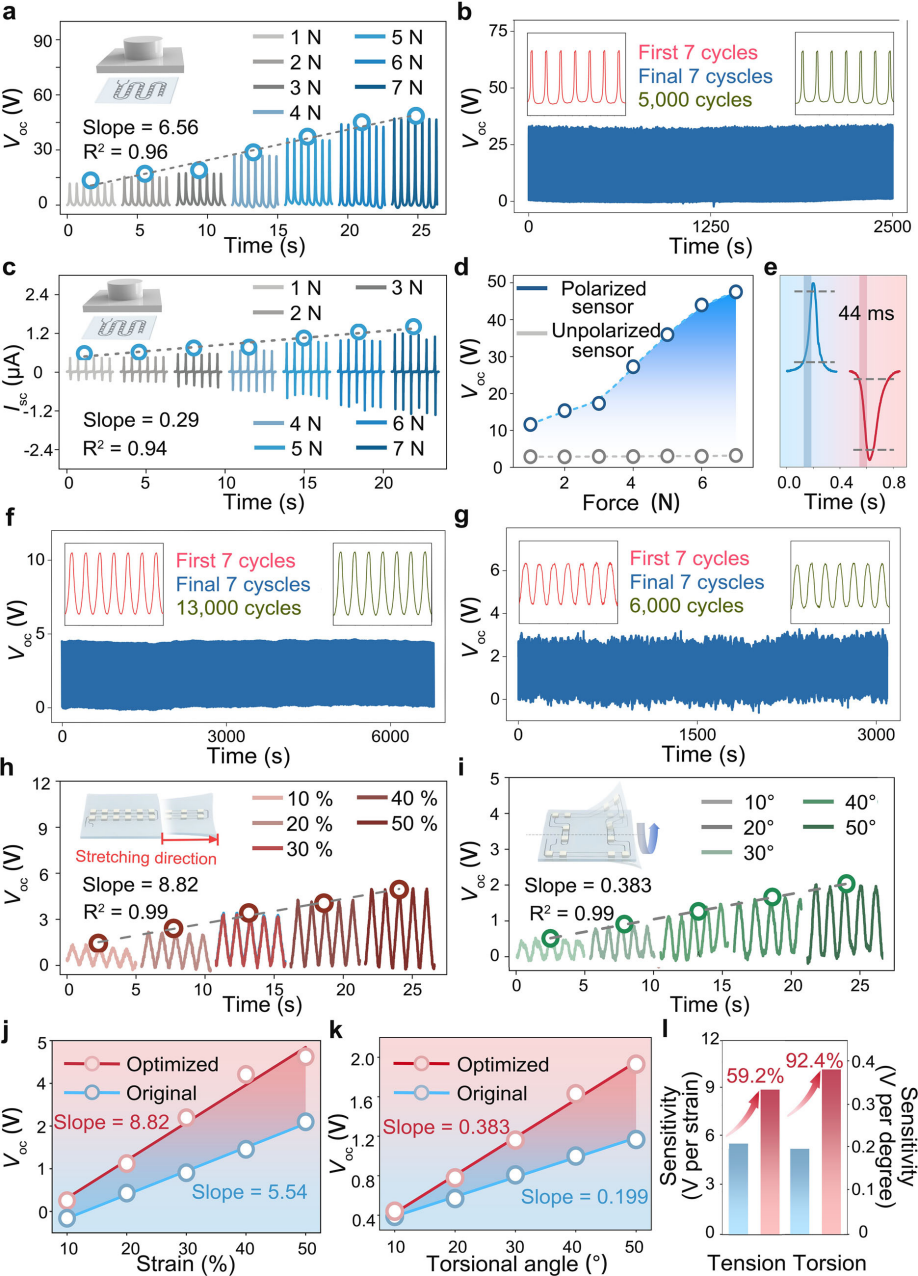
Electrical properties and optimization on stretchable sensor characteristics. When subjected to a compressive deformation, sensors exhibit a) changes of *V*_oc_ at 2 Hz for a range of forces, linearly increase in *V*_oc_ with force from 1 N to 7 N (inset: schematic of stretchable sensor subjected to compressive deformation) and b) high stability over 5,000 cycles at 4 N at 2 Hz. (inset: signal details of the first and final seven cycles). When subjected to compressive deformation, sensors also exhibit c) an increase in *I*_sc_ at 2 Hz for a range of forces and linearly increase in *I*_sc_. d) Comparison of *V*_oc_ between polarized and unpolarized sensors subjected to compressive deformation. e) *V*_oc_ and response time generated by the sensor in forward and reverse connections. f) During tensile deformation sensors exhibit excellent durability over 13,000 cycles at 40% strain at 2 Hz. g) Under torsional deformation sensors show good stability over 6,000 cycles at 90° strain and 2 Hz. (inset: signal details of the first and final seven cycles). h) During tensile deformation, optimized sensors exhibit variation of *V*_oc_ at 1 Hz for a range of strain, linearly increasing *V*_oc_ with strain from 10% to 50% (inset: schematic of stretchable sensor subjected to tensile deformation) i). Under torsional deformation, optimized sensors exhibit variation of *V*_oc_ at 1 Hz for a range of angles, linearly increasing *V*_oc_ with angle from 0° to 50° (inset: schematic of stretchable sensor subjected to torsional deformation). Comparison of *V*_oc_ between optimized and original sensor for j) tensile deformation and k) torsional deformation. l) Comparison of sensitivity between optimized and original sensor for tensile deformation and torsional deformation.

Due to the high voltage coefficient, *g*_33_, of the porous ceramics and the precisely tailored EGaIn electrode circuits, we fabricated a 3 × 5 array stretchable piezoelectric sensor with a high degree of freedom in mechanical deformation (Figures S26 and S27). The electrical performance of the stretchable piezoelectric sensor was carefully evaluated under three modes of deformation: compression, tension, and torsion. As shown in Figure 5a,d, the open‐circuit voltage (*V*_oc_) and short‐circuit current (*I*_sc_) increased with increasing applied pressure, indicating that the sensor has an excellent response to pressure stimulation, with maximum values of 47.4 V and 2.31 µA, respectively. Sensor sensitivity can be defined as the ratio of the difference between peak and valley voltage with strain [53, 54, 55, 56]. High sensitivities of 6.56 V N^−1^ (R^2^ = 0.96) and 0.29 µA N^−1^ (R^2^ = 0.94) were obtained over a wide range of stress and strain (Figure 5b,e). In addition, after 5,000 compression cycles at 2 Hz and 4 N (Figure 5c), the *V*_oc_ remains above 99% of the initial value, demonstrating that the sensor has high mechanical stability. The *V*_oc_ of the polarized sample was significantly higher than that of the unpolarized sample (Figure 5f), proving that the signal originated from the piezoelectric effect. Furthermore, by changing the forward connection and reverse connection of the oscilloscope, it was further confirmed that the output signal arose from the piezoelectric response induced by polarization changes (Figures S28, S29). By measuring the *V*_oc_ under forward and reverse connection during applied pressure, the sensor exhibited a response time of 44 ms (Figure 5g).

After obtaining encouraging results during compressive stimulation, the sensing response of the sensor when subjected to a tensile load was further quantitatively characterized. As the tensile strain increased from 10% to 60%, the *V*_oc_ increased from 1.77 V to 8.96 V (Figure S30), and the sensitivity was as high as 14.0 V per strain with a high linearity of 0.99 (Figure S31). Similar to the response under compression, the unpolarized sensor only exhibited a low level of voltage signal, thereby excluding the influence of triboelectric effects (Figure S32). In addition, the sensor was subjected to fatigue cycle testing during application of a 40% tensile strain at 2 Hz (Figure 5f), where the *V*_oc_ decreased by 2.6% after 13,000 cycles. The sensor was also tested for over 6,000 cycles when subject to a range of humidity conditions (Figures S33–S35), demonstrating excellent mechanical robustness and environmental adaptability. Compared to compression and tension, the application of torsion represents a more complex mechanical deformation mode for sensors, which requires both high mechanical flexibility and stretchability in the sensor design. Figure S36 shows the response of the sensor during torsion deformation ranging from 30° to 180° at 2 Hz. The *V*_oc_ exhibited a peak output of 5.54 V and a high sensitivity of 0.10 V per degree with linearity of 0.99 (Figure S37. The *V*_oc_ of the nonpolarized sensors is significantly lower than the polarized sensors (Figure S38), also excluding the influence of triboelectric effects. As shown in Figure 5g, after 6,000 cycles subject to 90° torsion, the sensors remained 95.5% of their initial *V*_oc_. Compared to previously reported works, the sensors also showed a high *V*_oc_ and durability (Table S11). The output voltage of the sensor fabricated based on the topology optimization strategy when subject to tensile and torsional deformation (Figure S39, S40) was systematically evaluated (Figure 5h,i). The experimental results demonstrated that the *V*_oc_ and sensing sensitivity of the sensor were significantly enhanced under both tensile and torsional deformation tests. As shown in Figure 5j,k and Figures S41, S42, when subject to strains ranging from 10% to 50% and a torsional angle range of 10° to 50°, the optimized sensor exhibited an increase of 57.0% and 44.5% in the average *V*_oc_, respectively (Movie S3). On comparing the sensitivity of the optimized and original sensor (Figure 5l), the optimized sensors exhibited a sensing sensitivity that was improved by 59.2% (from 5.54 V per strain to 8.82 V strain) during tensile deformation and by 92.4% (from 19.9 mV per degree to 38.3 mV per degree) during torsion, respectively. The experimental results were in good agreement with the simulation predictions, confirming the critical role of the topology optimization strategy in enhancing the output performance of the sensor. In addition, these results also demonstrate the excellent compatibility of tailored stretchable interconnects with the optimization strategy.

### 2.4 Monitoring Neck Motion

Motivated by the concept that topology optimization enables optimal stress utilization in stretchable piezoelectric sensors when subjected to anisotropic deformation, we further explored its potential for applications associated with complex strain/deformation, such as a biological system involving multiple motion modes. As one of the most flexible regions of the human body, the neck undergoes significant and dynamic surface deformations [57], which include extensive stretching and torsional strains during routine movements [58]. These anisotropic mechanical inputs pose a significant challenge for conventional sensors based on homogeneously distributed piezoelectric phases [59], which typically lack the spatial resolution and structural adaptability that are required to accurately capture localized strain variations and distinguish between different motion features.

**FIGURE 6:**
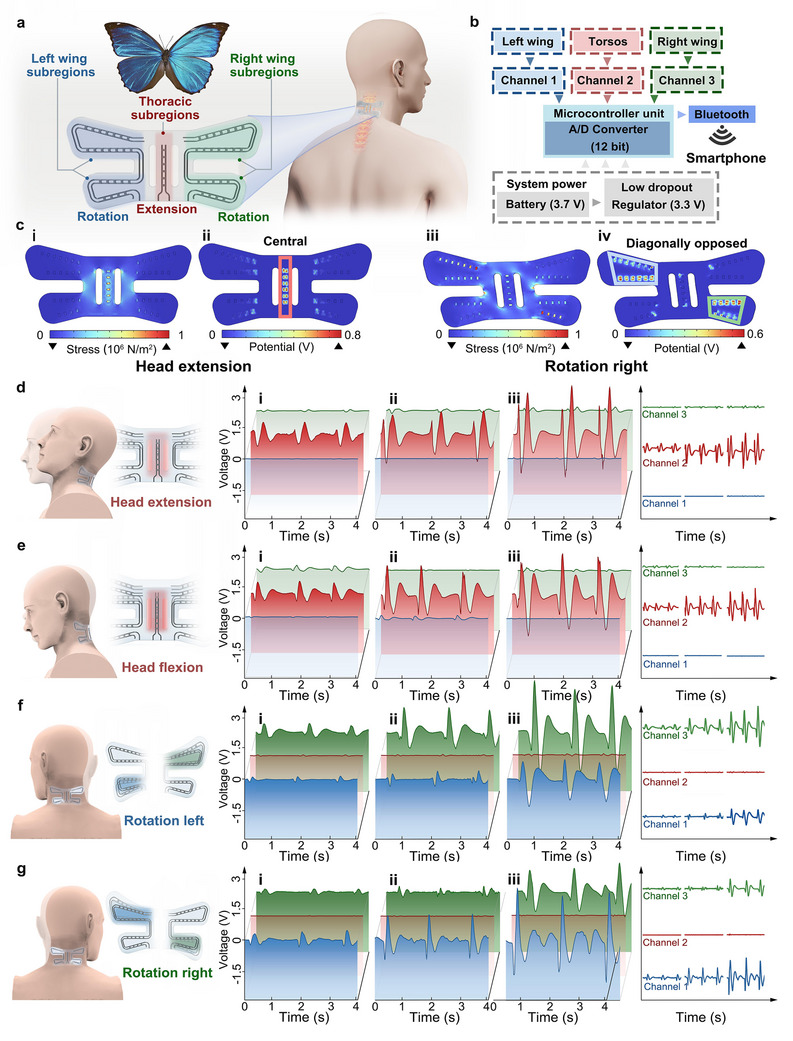
Structural design and application of a multi‐channel stretchable piezoelectric sensor. a) Schematic of a sensor inspired by butterfly structure for monitoring neck motion. b) System chart of the Bluetooth module, the three subregions of the sensor were connected to channels of a wireless module, and measured signals were transmitted via Bluetooth to a smartphone for real‐time display. c) FEA results of monitoring neck motion via sensors. During head extension, FEA results of (i) stress distribution and (ii) piezoelectric potential distribution, the potential is primarily concentrated in the central functional subregion (red box). During head rotation to the right, the FEA results of (iii) stress distribution and (iv) piezoelectric potential distribution, potential is primarily concentrated in diagonally opposed wing subregions (blue and green box). Output voltage of three channels of sensor during d) head extension, e) head flexion, f) rotation left, g) right rotation, and right at (i to iii) three ranges of motion amplitudes.

Inspired by the morphological characteristics of butterflies, we developed a multi‐channel stretchable piezoelectric sensor. This sensor is able to mimic the bilateral symmetry and functional partitioning of butterfly wings by employing a structure that combines aesthetics with mechanical performance. As shown in Figure 6a, the sensor is divided into three functional subregions‐central torsos, namely the left wing and right wing, which are similar to the thorax and the wings of a butterfly. Each functional subregion of the sensor is equipped with a region‐specific topology‐optimized layout, thereby enhancing the coupling of stress and sensing the independent mechanical response within each subregion. Notably, the wing‐like lateral regions further exhibit horizontal asymmetry: the lower section of each wing contains fewer piezoelectric units than the upper section, resulting in a lower piezoelectric output under equivalent strain conditions. This intentionally designed asymmetric structure enables the sensor to distinguish the direction of torsion based on the differential signal between the upper and lower subregions. The microcontroller unit collects the piezoelectric voltage generated by each region through three transmission channels and subsequently transmits it to a smartphone via Bluetooth signals, thereby enabling real‐time, anisotropic deformation acquisition (Figure 6b).

We first used FEA to analyze the stress distribution in each functional subregion of the sensor during four basic neck motion modes: (i) extension, (ii) flexion, (iii) left rotation, and (iv) right rotation. As shown in Figure 6c(i) and (ii), during extension and flexion, the stress is primarily concentrated in the central functional subregion, while the lateral wings exhibit negligible deformation and minimal mechanical activation. In contrast, during rotation, the sensor undergoes torsional deformation, which effectively redirects the stress toward the left‐ and right‐wing regions, resulting in significant piezoelectric response (Figure 6c(iii) and (iv)). Furthermore, consistent with the topology optimization analysis discussed in Section 2.1, the stress and piezoelectric potential generated during the torsional deformation are primarily concentrated in the diagonally opposed wing‐shaped sub‐regions of the butterfly‐shaped sensor. The resulting voltage‐time curves during different neck motions further confirm the unique ability of the stretchable bioinspired butterfly‐shaped sensor to identify motion signatures. As shown in Figure 6d(i), during head extension, the central functional subregion (Channel 2) exhibits a significant output voltage (1.20 V), while the wing‐like lateral subregions (Channels 1 and 3) generate minimal responses (0.11 V and 0.01 V), indicating that tensile strain is primarily concentrated in the central region, consistent with the initial design. From Figure 6d(i–iii), it can be seen that as the extension amplitude increases, the output voltage of Channel 2 increases significantly from 1.21 V to 4.17 V, while Channels 1 and 3 remain below 0.28 V, confirming the sensor's capacity for both the modes of deformation recognition and amplitude resolution. A similar trend was observed during head flexion, which produces a characteristic signal inversion relative to extension, accompanied by a similar spatial stress distribution (Figure 6e).

Figure 6f,g further demonstrate the sensor's ability to capture the dynamics of neck rotation. Consistent with the simulation results, leftward or rightward rotation of the neck selectively activates Channels 1 and 3, which correspond to the lateral wing regions, while Channel 2 corresponds to the central region. During leftward head rotation, the output voltage of the left wing (Channel 1) is consistently lower than that of the right wing (Channel 3); the opposite trend is observed for a rightward rotation. This capability for directional recognition arises from the sensor's deliberately engineered horizontal asymmetry. When subjected to torsional deformation, the mechanical stress is primarily concentrated in diagonally opposed wing subregions, thereby activating the contralateral upper wing and the ipsilateral lower wing, respectively. Since the upper wing has a higher density of piezoelectric units than the lower wing, the contralateral side consistently generates a higher piezoelectric response, thereby enabling reliable identification of head rotation direction based on the relative peak output voltages. The above results show that the topology‐optimized multi‐channel stretchable piezoelectric sensor exhibits excellent regional decoupling ability, stress‐specific response ability, and reliable neck motion recognition, demonstrating its significant application potential in fields such as multi‐dimensional human motion monitoring and human‐computer interaction technology.

## 3 Conclusions

This paper has developed a topology‐optimization strategy to create a stretchable piezoelectric sensor which integrates flexible electrode circuits based on a eutectic gallium‐indium (EGaIn) liquid metal and porous piezoelectric ceramic units. This new strategy significantly enhances the electro‐mechanical adaptability and efficient utilization of the spatial stress distribution within the sensor system. Finite element analysis demonstrates that, compared to an unoptimized sensor, the maximum piezoelectric potential, *φ*_max_, developed by the topology‐optimized sensor increased by 103.5% under tensile deformation and 59.7% under torsional strain. To fulfill the dual requirements of controlled manufacturing and achieving high mechanical stretchability of the electrodes, we established an analytical model of the EGaIn extrusion dynamics during direct ink writing, achieving 94.7% accuracy in predicting the printing state and a relative error of less than 15% in trace width, *w*. The printed electrode circuits demonstrated exceptional electromechanical durability, maintaining a stable electrical conductivity over 22,000 tension cycles at 60% strain. By electrically connecting high‐performance porous piezoelectric ceramic units arranged according to topology optimized assembly to the stretchable circuit, we fabricated a stretchable piezoelectric sensor that exhibited high mechanical stretchability, robustness, and sensing performance. The optimized sensor achieved a high sensitivity when subjected to both tensile (14.0 V per strain) and torsional deformation (0.10 V per degree). Compared to the unoptimized device, the sensor fabricated via topology‐guided assembly exhibits a 59.2% and 92.4% improvement in sensitivity under tensile and torsional modes, respectively. These results highlight the effectiveness of topology optimization in enhancing electromechanical responses under distributed load. Based on the optimized structure and circuit framework, we further developed a multi‐channel stretchable piezoelectric sensor inspired by the morphological features of butterfly wings and optimized according to our new approach. The biomimetic design was able to mimic the bilateral symmetry and functional partitioning of the butterfly morphology, enabling reliable identification of the type and magnitude of neck movements.

This work provides a new approach for the design and manufacture of high‐performance stretchable piezoelectric sensors by leveraging topology optimization, tailored EGaIn electrode printing, and porous ceramic engineering to simultaneously enhance mechanical durability, stretchability, and sensing sensitivity to produce highly deformable sensors capable of monitoring complex and anisotropic deformation in applications related to medical monitoring, biomechanical capture, wearables and high strain sensor networks.

## 4 Experimental Section

### 4.1 Computer‐Aided Topology Optimization

Topology optimization was undertaken using SolidWorks, where 12 cubic PZT piezoelectric ceramic units (2 mm × 2 mm × 2mm) and a silicone rubber polymer matrix (26 mm × 26 mm × 2.5 mm) were constructed. The piezoelectric units were embedded within the polymer matrix, using topology optimization constraints and a target defined. With the optimization target for maximizing the stress distribution on the active piezoelectric units, the automatic iterative calculations were performed in the SolidWorks Simulation Premium plugin, obtaining all 84 calculation result models. The model was imported into COMSOL Multiphysics 6.0 to validate the stress distribution and analyze the piezoelectric potential generated from the applied stress (Table S12). The materials and structural parameters were set according to the theoretical data and experimental conditions provided by the supplier (Tables S13 and S14).

### 4.2 Direct Ink Writing (DIW) of EGaIn Circuit Printing and Measurement

A eutectic gallium‐indium (EGaIn) Sigma‐Aldrich (Cat. No. 495425) was printed using a 3D printer (JDX02, Nayi Technologies, China). Oxide‐free EGaIn was loaded into a syringe equipped with a 25G nozzle, which was installed on a motorized stage. The printing parameters were: printing speed (*v*), flow rate (*Q*), and printing height (*h*). After generating G‐code to define the printing paths, the 3D printer program then printed the EGaIn circuits. Printed circuits were observed and evaluated using an optical microscope (BX53‐P, OLYMPUS, Japan). The electrical resistance of the printed circuit under different tensile strains was recorded by a digital multimeter (DMM7510, Keithley, USA).

### 4.3 DIW of EGaIn: Mechanism of Printing and Analysis

Based on the dynamic analysis of the EGaIn extrusion process and the regulation between shear stress (*τ*) and yield stress (*σ*), the governing equations were derived. Although a dense oxide layer forms on the surface of EGaIn, this oxide film suppresses further oxidation, and the inner liquid EGaIn can still be regarded as a Newtonian fluid, which was suitable for applying Newtonian fluid shear force models [60]. Accordingly, shear stress at the nozzle and the substrate can therefore be defined as:

(7) $$\tau_{\mathrm{nozzle}}=\eta \frac{v}{t-h}$$

(8) $$\tau_{\mathrm{substrate}}=\eta \frac{v}{h}$$

where *v* was the printing speed, *Q* was the flow rate. *η* was the viscosity of EGaIn, *h* was the printing height, and *t* was the trace thickness. As shown in Figure S10, based on the geometric relationships, the cross‐sectional area of the trace can be calculated by the elliptical formulation as:

(9) $$S=\pi ab\left(\theta -\sin\theta \;\cos\theta \right)$$

where *a* and *b* were the semi‐minor and semi‐major axes of the ellipse, respectively. For a contact angle *θ* of the EGaIn was 150.2° (Figure 3e), the ratio of the cross‐sectional area between the arc‐ellipse segment and the full‐ellipse was 0.97. Due to the large and nearly constant contact angle, the area can be equivalently considered as a full‐ellipse [61]. As shown in Figure S11, with the trace width*, w =* 2*b*, and the trace thickness, *t =* 2*a*, the area of this ellipse can be expressed as:

(10) $$S=0.97\frac{k\pi w^{2}}{4}$$

where, aspect ratio was defined as *k* (*k = t/w*). Then, the corresponding EGaIn volume, 𝑉, within per unit length, 𝐿_0_, can be calculated via:

(11) $$V=Q\;\frac{L_{0}}{v}=0.97\frac{k\pi w^{2}}{4}L_{0}$$

and *t* can be determined by Equation (12):

(12) $$t=\sqrt{\frac{4kQ}{0.97\pi v}}\;$$

According to the geometrical relationship between the droplet surface and the direction of shear stress (Figure 3d), the tangential components of shear stress along the droplet surface at the nozzle and substrate, denoted as *τ*_1_ and *τ*_2_, were expressed as Equations (13) and (14).

(13) $$\;\tau_{1}=\tau_{\mathrm{nozzle}}$$

(14) $$\;\tau_{2}=\tau_{\mathrm{substrate}}\cdot \cos\left(\pi -\theta_{\mathrm{adv}}\right)$$

By substituting the tangential components into the governing relations, Equations (3) and (4) were obtained. The viscosity of the EGaIn, *η*, was 1.99 mPa·s [41]. As measured in Figure 3e, the advancing contact angle (*θ*_adv_) of the EGaIn was 156.2°. Boley et al. [62]. obtained the cross‐sectional dimensions of the printed trace under different parameters, which implied a change in *k* that was dependent on *h*. Subsequently, the calculation indicates that when h = 0.01 mm, 0.02mm, 0.03 mm, the parameter k = 0.67, 0.76 and 0.80, respectively. By solving the corresponding Equations (3) and (4) with these parameters, the values of the shear stress components *τ*_1_ and *τ*_2_ were obtained (Tables S1 and S2). Due to the thickness of the oxide layer of 1–5 nm, the shear stress was equivalent to the surface stress after neglecting the stress in the thickness direction [63, 64]. By comparing the calculated shear stress with the theoretical yield stress, the printing states when subjected to different printing parameters were predicted.

Equation (5) for the trace width, *w*, can be obtained after formula simplification of Equation (11). By solving Equation (5) for different parameter conditions, the predicted trace width (𝑤_pre_) was obtained (Table S3). The predicted results were compared with the experimental trace width (*w*_exp_) (Table S4) for error analysis, and the relative error (*ε*) was calculated via:

(15) $$\varepsilon =\frac{w_{\mathrm{pre}}-w_{\exp}}{w_{\mathrm{pre}}}\;$$

Data for calculated relative error, *ε*, results were shown in Table S5.

### 4.4 Fabrication of the Stretchable Piezoelectric Sensor

Lead zirconate titanate (PZT) ceramics with a random distribution of pores were synthesized by a process of sacrificial templating of polymer sphere. Polymethyl methacrylate (PMMA spheres, diameter = 20 µm) pore‐forming agents and PZT powder (PZT‐51 powder, Yu Hai Electronic Ceramics Co., Ltd.) were dispersed in water by magnetic stirring on a hotplate (300 rpm, 4 h), followed by the addition of a polyvinyl alcohol (PVA, 5 wt. %) binder and granulation. The mixed slurry was dried at 100 °C for 4 h. The powder obtained by granulation was poured into a square mold (20 mm × 20 mm) for pressing, and the bulk PZT ceramic materials were sintered at 1250 °C for 2 h, and then cut into a 2 mm thick ceramic sheet. After coating the bottom surface of ceramics with a thin layer of electrodes (room temperature conductive silver paste), the random porous ceramic sheets were subjected to corona polarization at room temperature for 30 minutes using a DC voltage of 19.5 kV to align ferroelectric domains along the direction of the electric field. Then, coating another electrode layer on the opposite side of the ceramic, the polarized ceramic sheet was cut into 2 mm × 2 mm × 2 mm units. The piezoelectric ceramic units were assembled with correspondingly sized stretchable conductive EGaIn circuits to ensure that the ceramic electrodes maintained their electrical connectivity. After comparing the mechanical properties of representative encapsulation materials, including PDMS and TPU (Table S15), Ecoflex 00–30 silicone elastomer (Ecoflex 00–30, Smooth‐on Inc, with 10 wt. % PDMS added) was used for encapsulation, which was cured at 70 °C temperature for 1 h, with the PDMS additive enhancing interfacial adhesion to the PZT units [12] (Figure S26).

### 4.5 Material Characterization

Pore morphology and distribution were analyzed by field‐emission scanning electron microscopy (FESEM, NovaNanoSEM230, USA). Porosity levels were measured using the Archimedes method. Dielectric properties (relative permittivity and dielectric loss) were measured from 1 kHz to 10 MHz, using a precision impedance analyzer (4294A; Agilent Technologies, Santa Clara, USA). Polarization‐electric field (*P‐E*) hysteresis loops were acquired by TF Analyzer 2000 (aixACCT systems, Germany). The piezoelectric charge coefficient (*d*_33_) of random porous PZT ceramics was determined by the piezoelectric *d*_33_ meter (ZJ‐4AN, Institute of Acoustics, Academic Sinica, China).

### 4.6 Measurement

A series of compressive, tensile, and torsional loads were applied to the sensors at a controlled frequency using a programmable linear motor to systematically evaluate the electrical performance of the stretchable piezoelectric ceramic sensors. An electrometer (6517B, Keithley, USA) was used to measure the open‐circuit voltage (*V*_oc_) and short‐circuit current (*I*_sc_) of the sensor.

### 4.7 Monitoring Neck Motion

The study was approved by the Ethics Review Committee of Xiangya Hospital, Central South University. The neck motion monitoring sensor was fixed to the posterior region of the neck of participants using medical‐grade adhesive tape. A nanogenerator analyzer (T5000‐18c, Beijing Huyi Technology Company, China) was connected to three channels of the sensor. Participants were instructed to perform four actions at a frequency of ≈ 0.5 Hz under a range of amplitudes: head extension, head flexion, head rotation left, and head rotation right. Three‐channel synchronous signals were recorded through the nanogenerator analyzer.

## Conflicts of Interest

The authors declare no conflict of interest.

## Supporting information




**Supporting File 1**: adma72410‐sup‐0001‐SuppMat.docx.

**Supporting File 2**: adma72410‐sup‐0002‐MovieS1–S3.zip.

## Data Availability

The data that support the findings of this study are available from the corresponding author upon reasonable request.
