# Supplementary material for: Topology‐Optimized Stretchable Piezoelectric Sensors With Tailored Liquid‐Metal Circuits for Anisotropic Stress‐Adaptive Motion Monitoring
Source: Adv Mater. 2026 Feb 7;38(15):e18168. doi: 10.1002/adma.202518168 (PMC12983431; doi:10.1002/adma.202518168)
Supplement: Supplementary file 1 — Supporting File 1: adma72410‐sup‐0001‐SuppMat.docx. [file ADMA-38-e18168-s002.docx]

Supporting Information

**Topology-Optimized Stretchable Piezoelectric Sensors with Tailored Liquid-Metal Circuits for Anisotropic Stress-Adaptive Motion Monitoring**

*Hanmin Zeng, Qianqian Xu***, Jianxun Zhang, Peiqiong Zhou, Jiachen Zhang, Jinlan Li, Senfeng Zhao, Kechao Zhou, Dou Zhang, Chris Bowen*, Yan Zhang**

H. Zeng, Q. Xu, J. Zhang, P. Zhou, J. Zhang, J. Li, Prof. K. Zhou, Prof. D. Zhang, Prof. Y. Zhang

State Key Laboratory of Powder Metallurgy, Central South University, Changsha, Hunan, 410000, China.

E-mail: qianqianxu@csu.edu.cn, yanzhangcsu@csu.edu.cn

1. Xu

Department of Mechanical and Acrospace Engineering, Hong Kong University of Science and Technology, Clear Water Bay, Hong Kong SAR, 999077, China.

S. Zhao

Hunan Provincial Key Laboratory of Micro & Nano Materials Interface Science, College of Chemistry and Chemical Engineering, Central South University, Changsha, Hunan, 410083, China.

Prof. C. Bowen

Department of Mechanical Engineering, University of Bath, Bath, BA2 7AY, UK.

E-mail: c.r.bowen@bath.ac.uk

**This PDF file includes:**

Figures S1 to S42 and Table S1 to S15.

**Other Supporting Online Information for this manuscript**

Movie S1 to S3.

**Supplementary figures and tables**

**
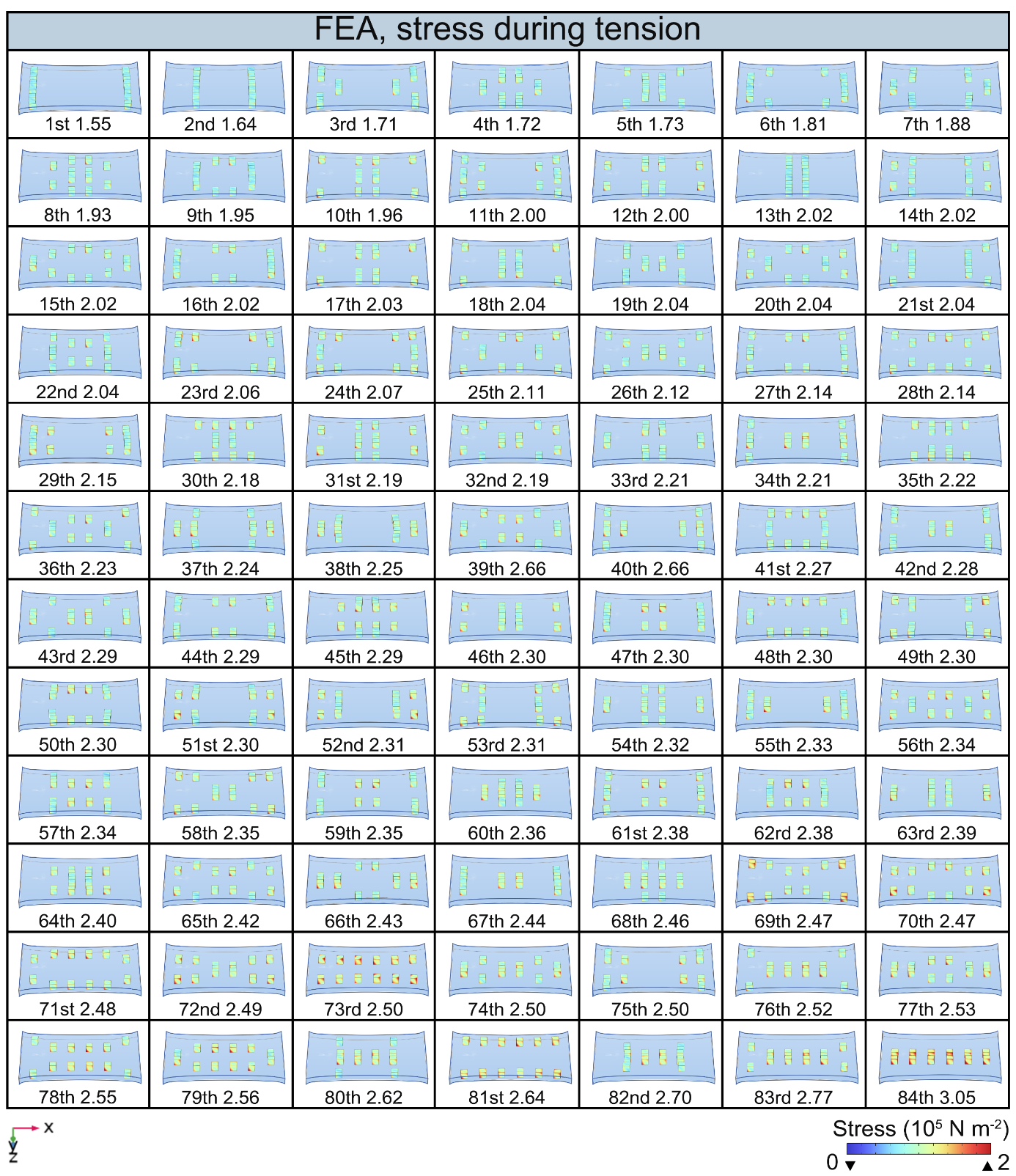
**

**Figure S1.** 84 Finite element analysis (FEA) Von Mises stress (N m^-2^) results of topological iteration optimization during tensile deformation.


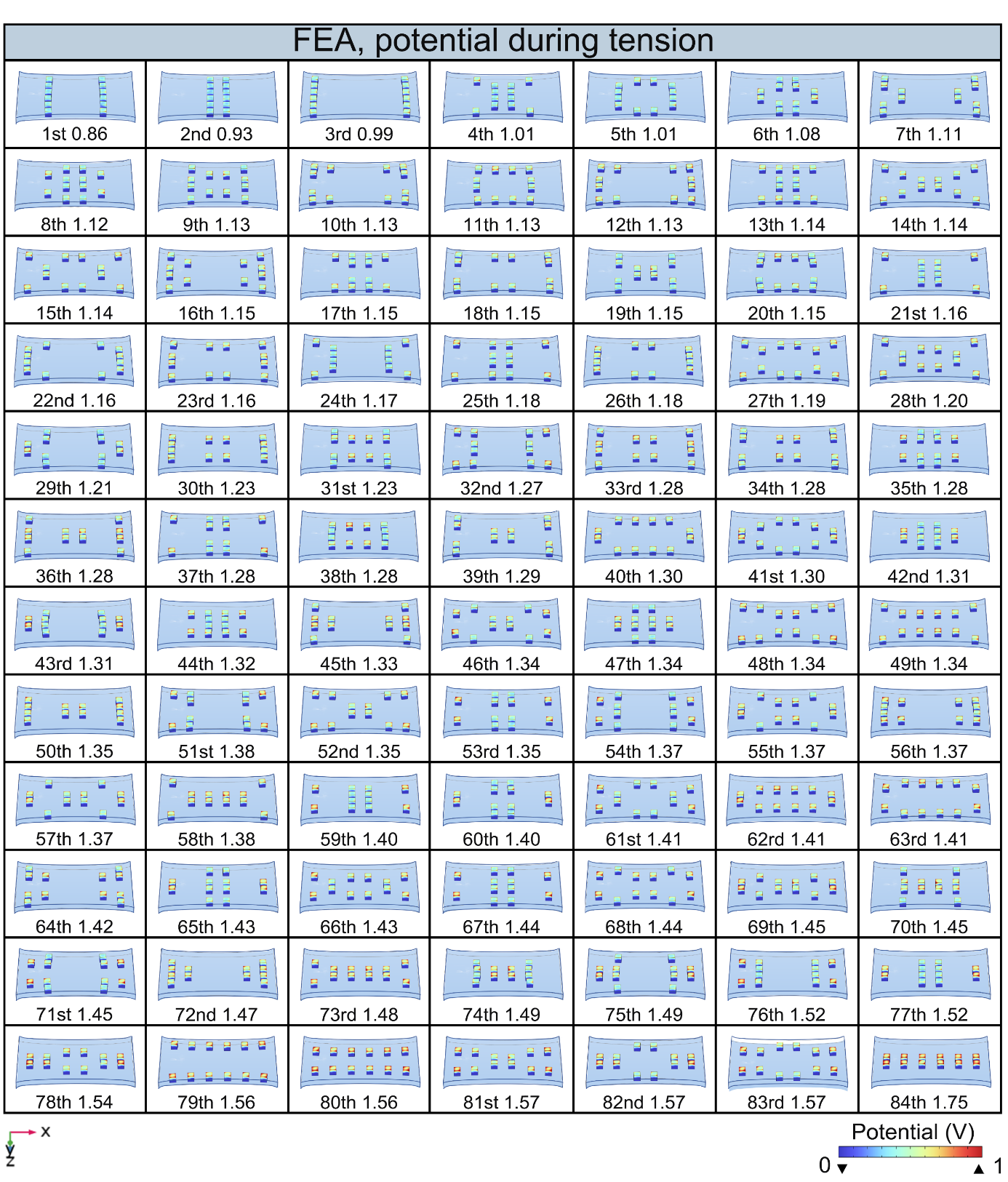


**Figure S2.** 84 FEA electrical potential (V) results of topological iteration optimization during tensile deformation.


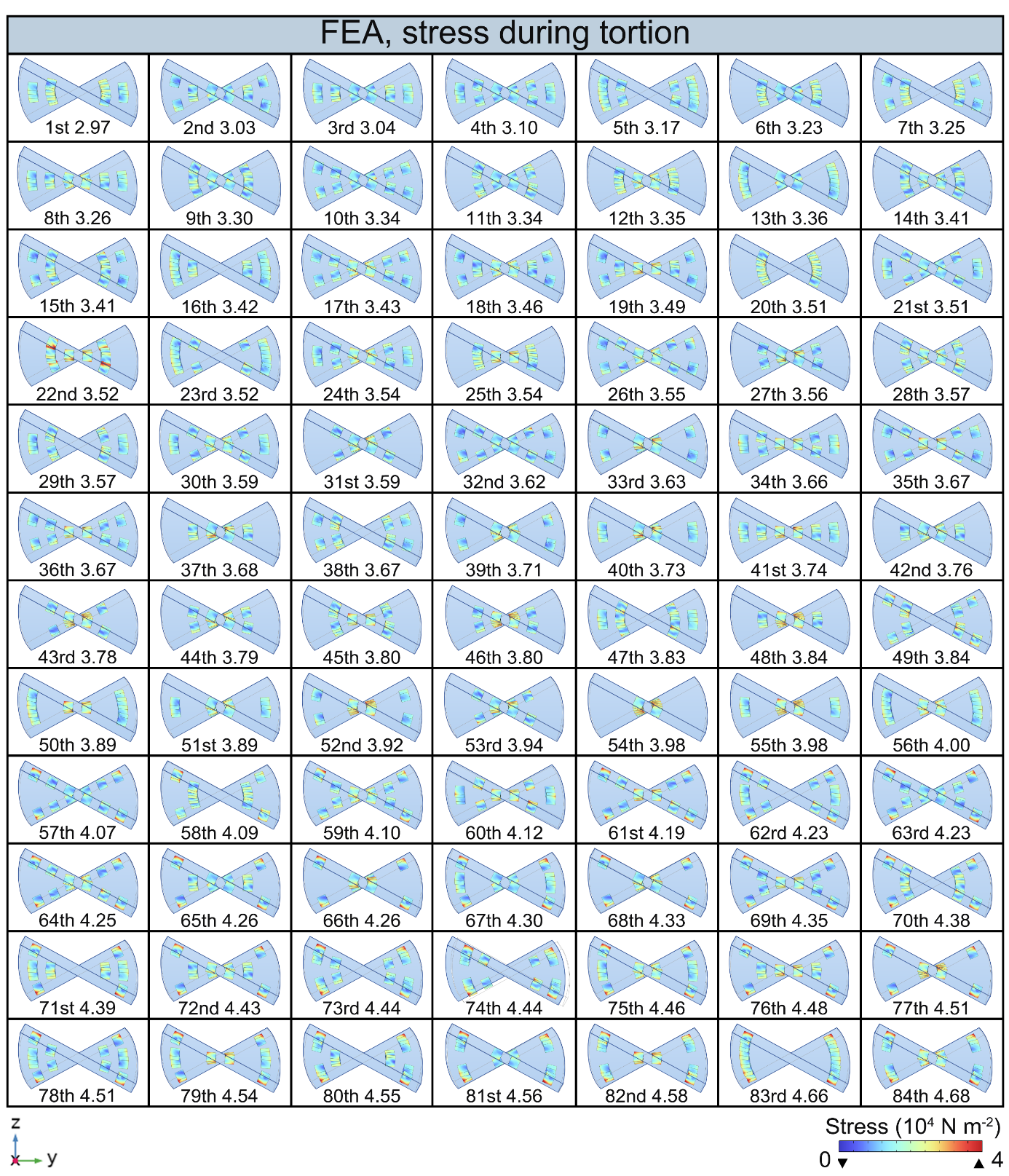


**Figure S3.** 84 FEA Von Mises stress (N m^-2^) results of topological iteration optimization during torsional deformation.


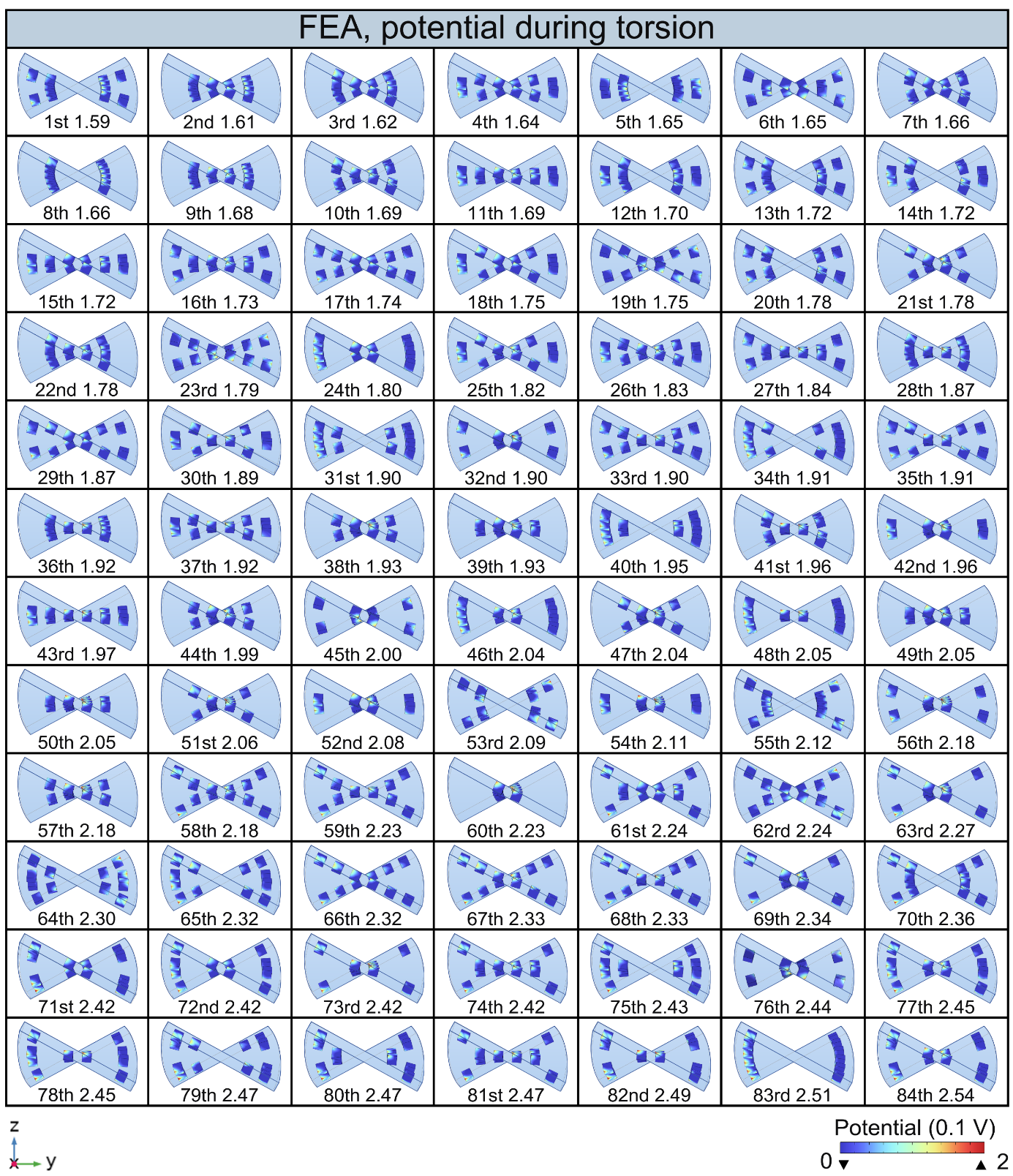


**Figure S4.** 84 FEA electrical potential (V) results of topological iteration optimization during torsional deformation.


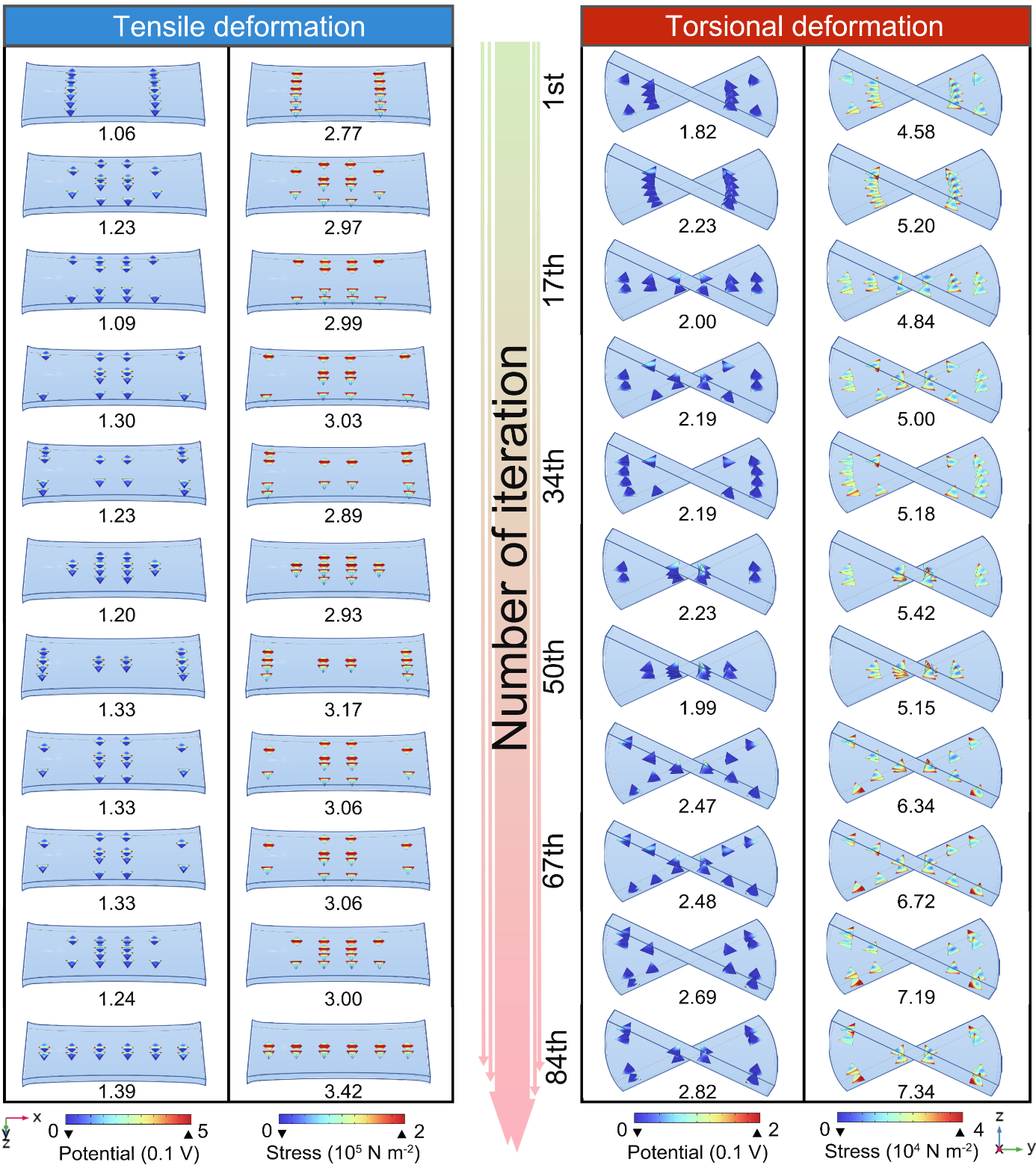


**Figure S5.** FEA results of stress and piezoelectric potential distributions for tetrahedral piezoelectric units obtained at the 1st, 8th, 17th, 25th, 34th, 42nd, 50th, 59th, 67th, 76th and 84th iterations subjected to tensile and torsional deformation.

**
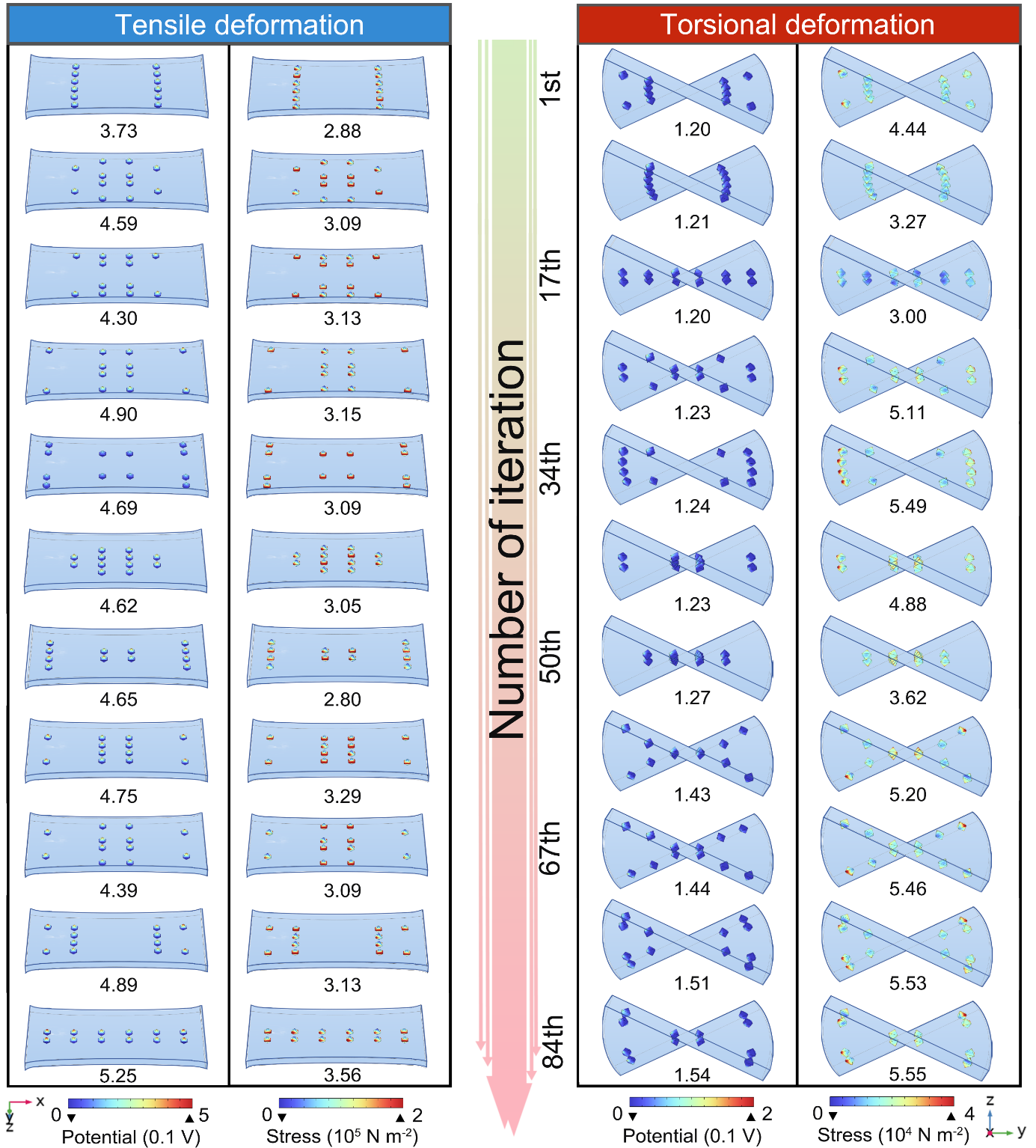
**

**Figure S6.** FEA results of stress and piezoelectric potential distributions for octahedral piezoelectric units obtained at the 1st, 8th, 17th, 25th, 34th, 42nd, 50th, 59th, 67th, 76th and 84th iterations subjected to tensile and torsional deformation.

**
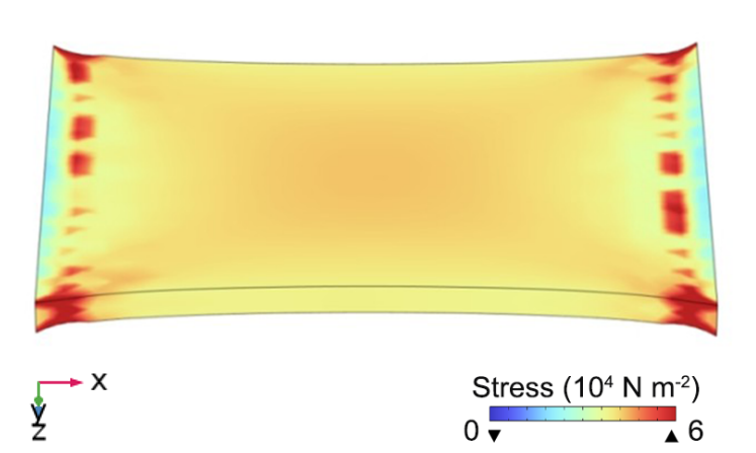
**

**Figure. S7.** FEA of Von Mises stress in the Ecoflex elastomer when subjected to tensile deformation.

**
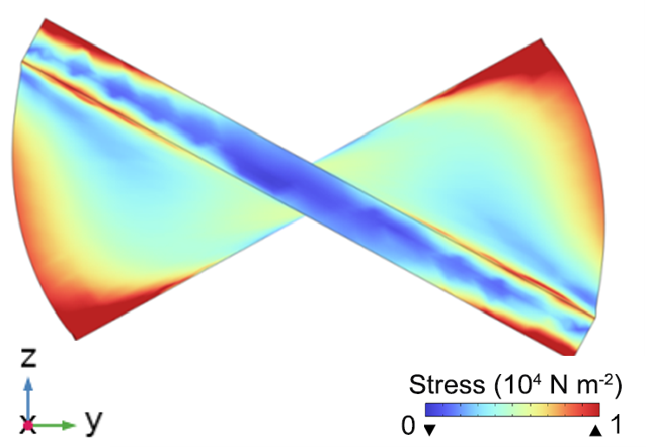
**

**Figure S8.** The FEA Von Mises stress of the Ecoflex elastomer when subjected to torsional deformation.


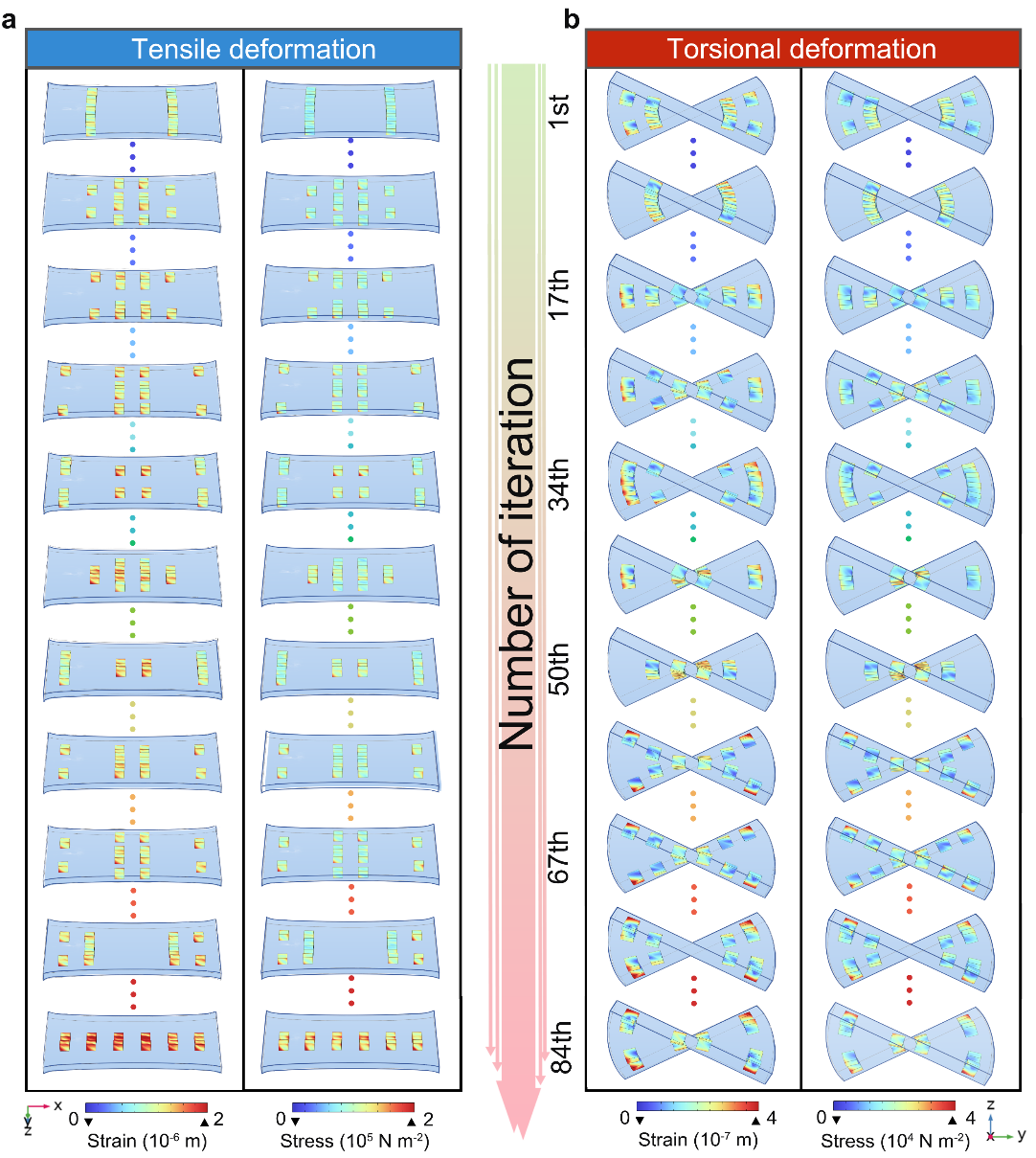


**Figure S9.** FEA results of strain and stress distributions for cubic piezoelectric units obtained at the 1st, 8th, 17th, 25th, 34th, 42nd, 50th, 59th, 67th, 76th and 84th iterations subjected to tensile and torsional deformation.


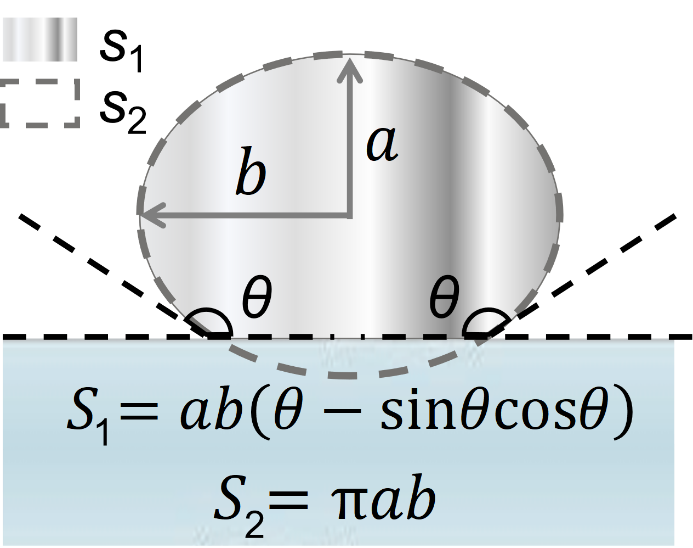


Figure S10. Geometrical relationship of the cross-sectional area between the arc-ellipse segment, *S*_1_, and full-ellipse, *S*_2_.


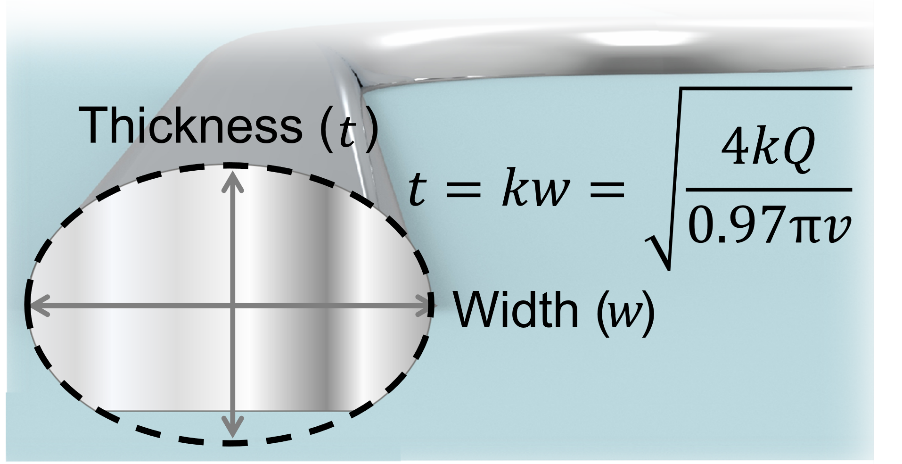


Figure S11. Geometrical and algebraic relationship between printed traced thickness, *t*, and printed traced width, *w*.


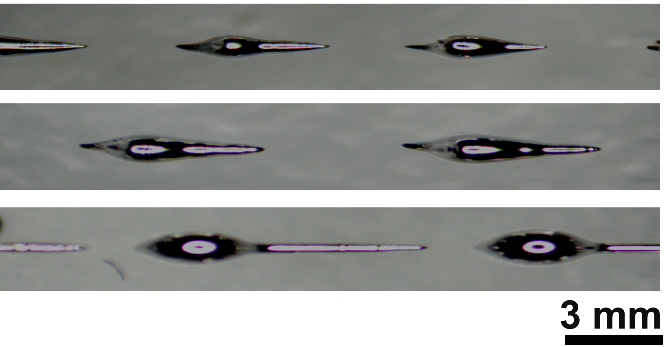


**Figure S12.** Optical image of the printing line in a ‘disconnection’ state.


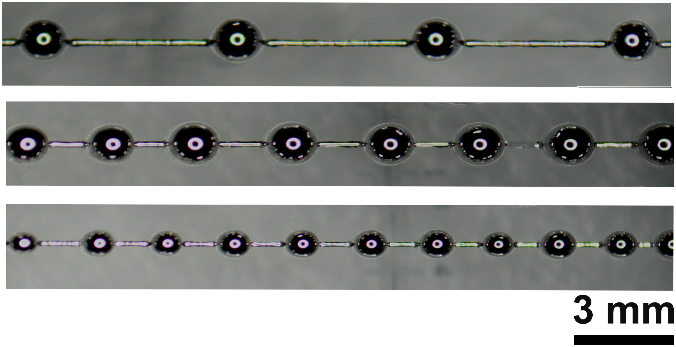


**Figure S13.** Optical image of the printing line in a ‘beading’ state.


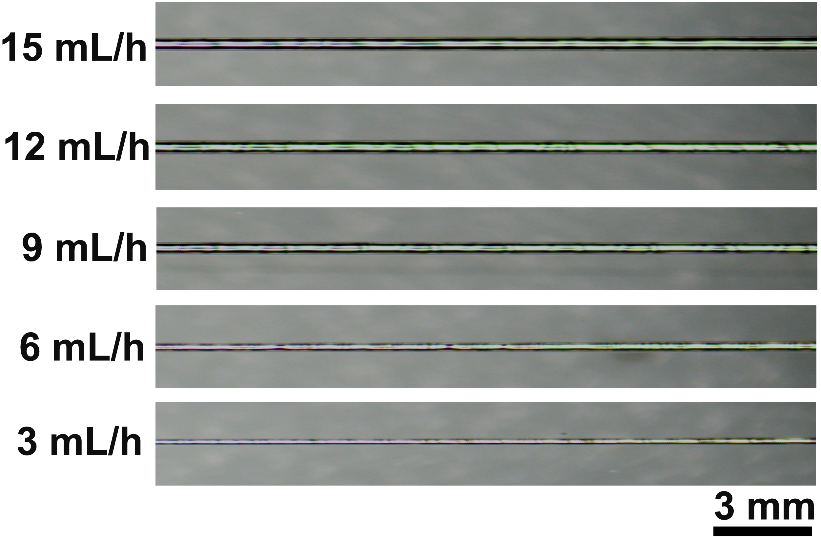


**Figure S14.** Optical image of the printing line under a *v* of 2 m min^-1^ and a *h* of 0.02 mm.


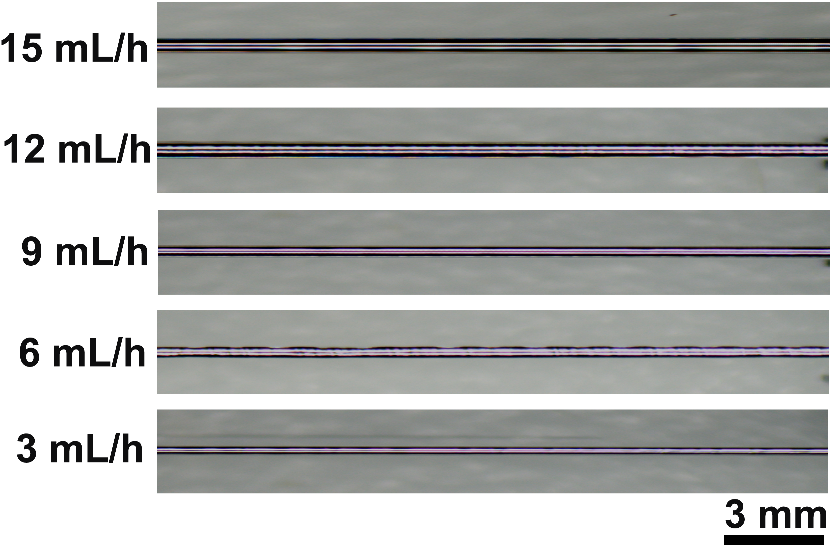


**Figure S15.** Optical image of the printing line under a *v* of 1.6 m min^-1^ and a *h* of 0.02 mm.


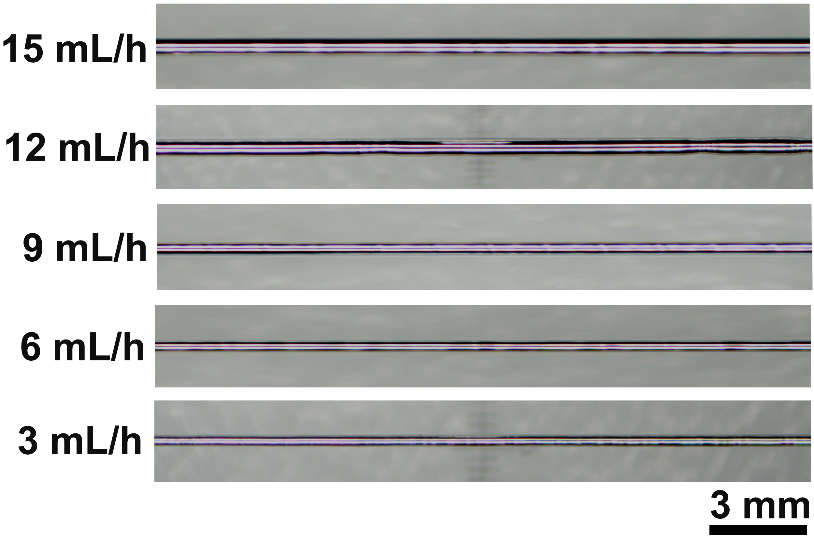


**Figure S16.** Optical image of the printing line under a *v* of 1.2 m min^-1^ and a *h* of 0.02 mm.


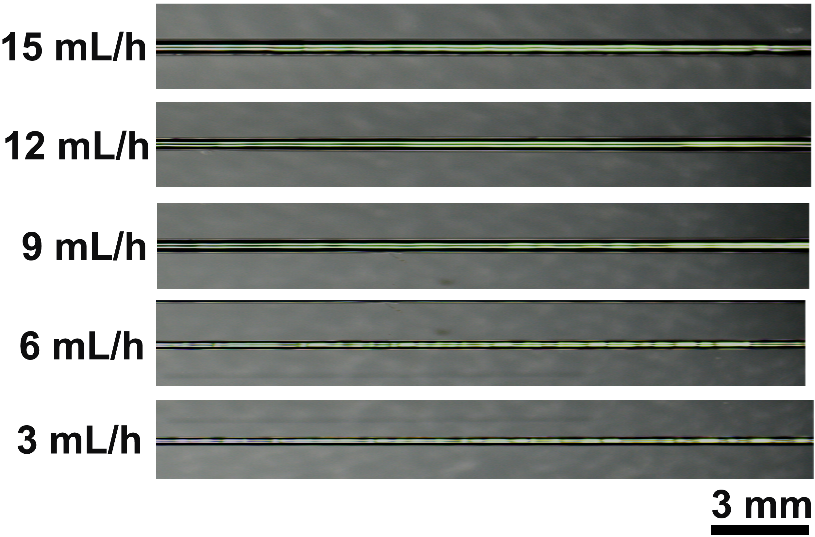


**Figure S17.** Optical image of the printing line under a *v* of 0.8 m min^-1^ and a *h* of 0.02 mm.


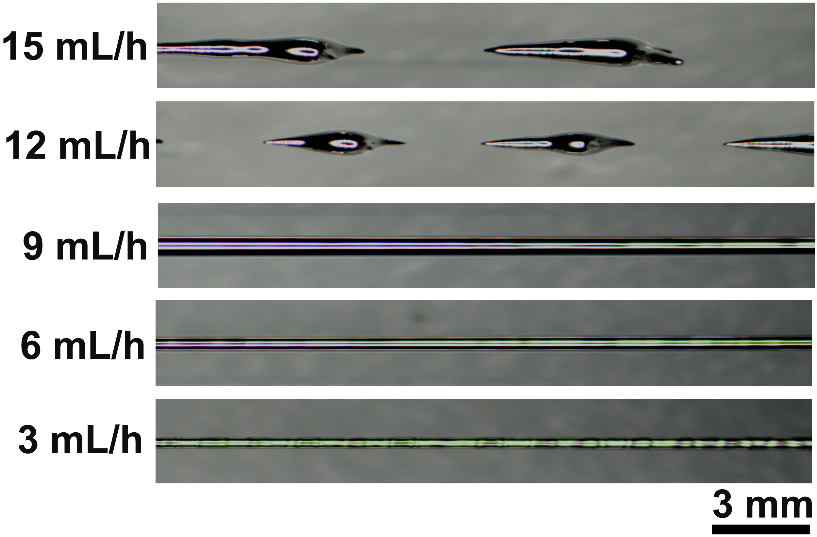


**Figure S18.** Optical image of the printing line under a v of 0.4 m min^-1^ and a h of 0.02 mm.


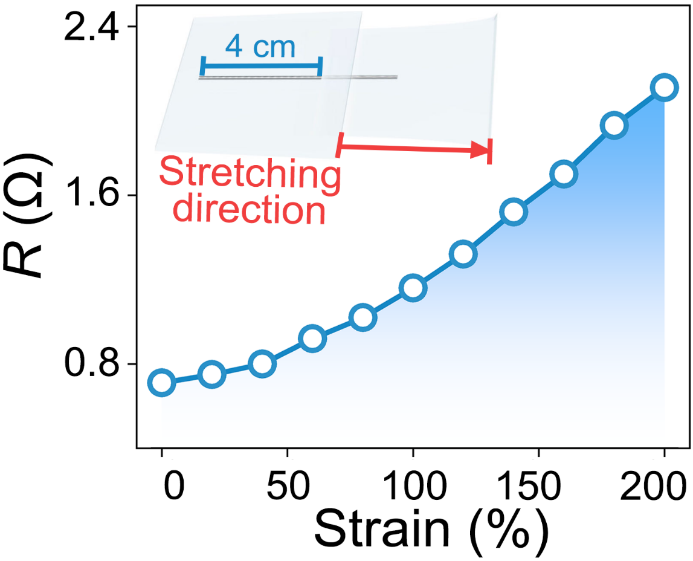


**Figure S19.** Resistance of a 0.1 mm *w*, 4 cm long EGaIn straight line subjected to a maximum strain of 200%.


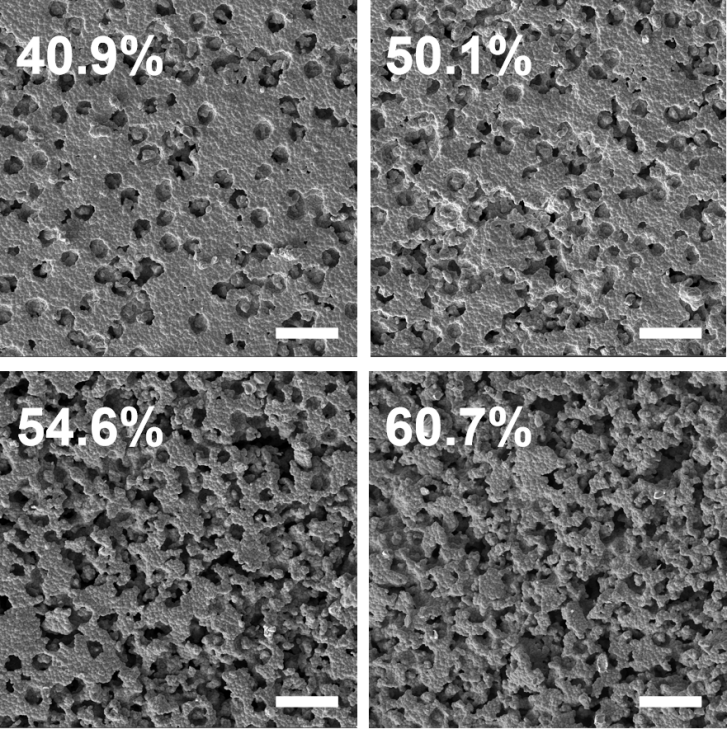


Figure S20. SEM images (scale bar = 50 μm) of random porous lead zirconate titanate (PZT) piezoelectric ceramics with porosity volume fractions of 40.9%, 50.1%, 54.6%, 60.7%, respectively.


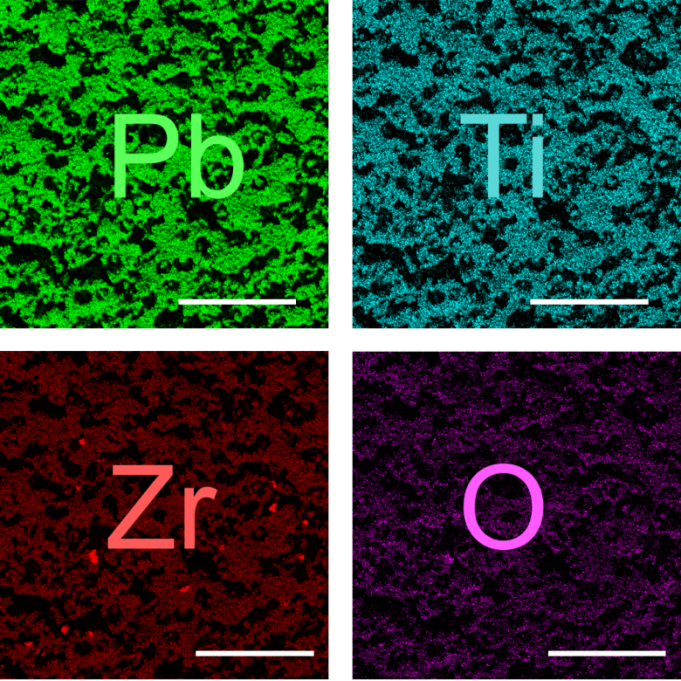


**Figure S21.** Energy dispersive spectroscopy (EDS) mapping of porous PZT ceramic with a porosity fraction of 60.7 vol% (scale bar = 100 μm).


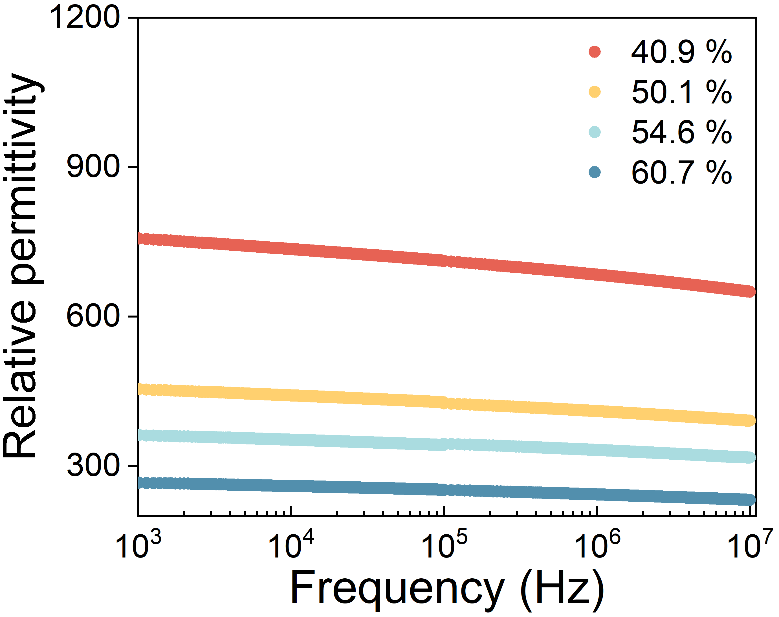


Figure S22. Relative permittivity of porous PZT ceramic for a range of pore volume fractions.


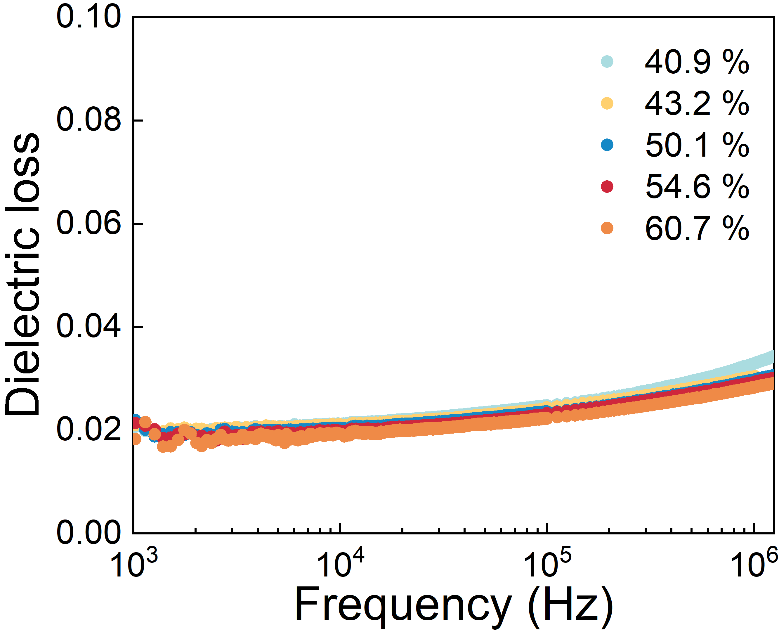


**Figure S23.** Dielectric loss of 3D lamellar structured PZT ceramic with porosity volume fractions of 40.9%, 50.1%, 54.6%, and 60.7%, respectively.


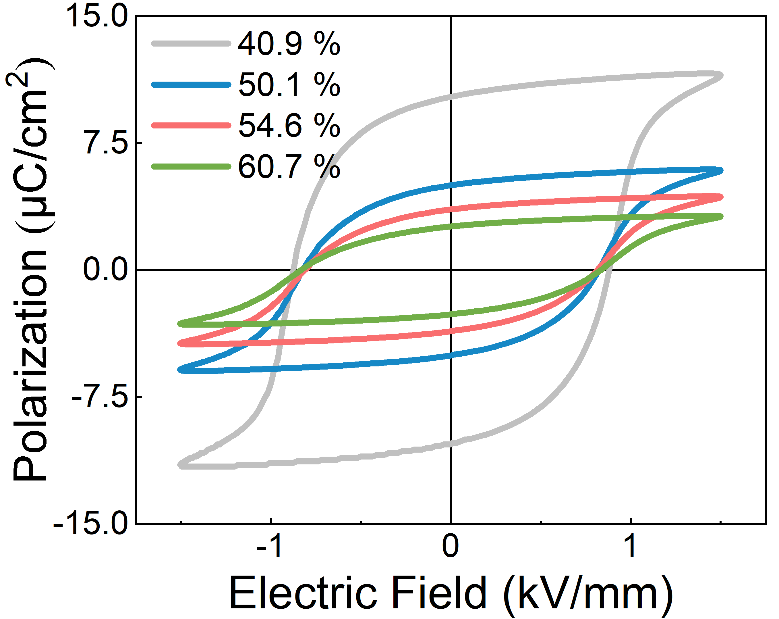


Figure S24. Polarization–electric field (*P-E*) hysteresis loops of porous PZT ceramic with a range of pore volume fractions.


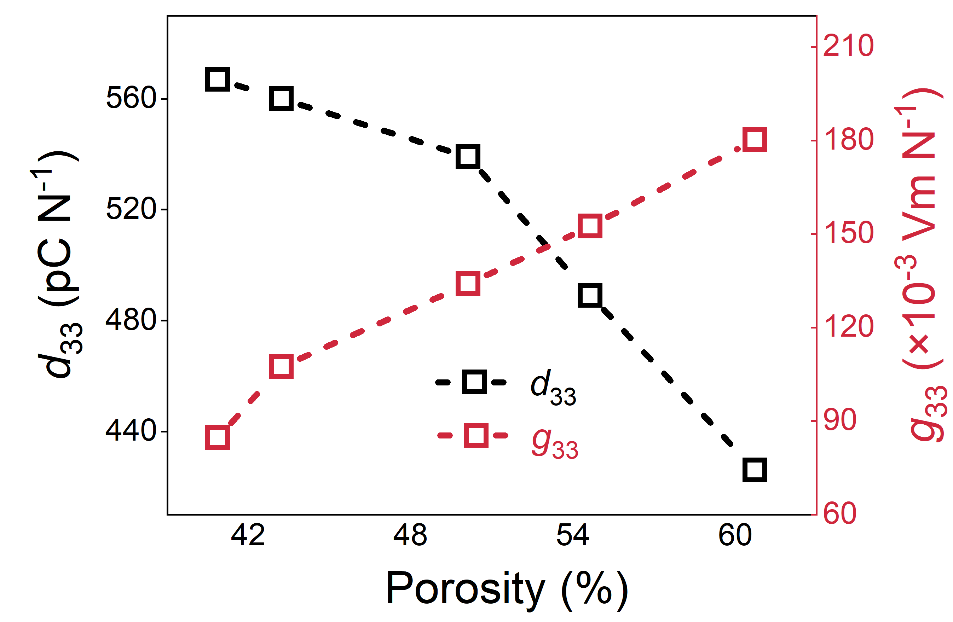


Figure S25. Variation of piezoelectric coefficient, *d*_33_, and piezoelectric voltage coefficient, *g*_33_, piezoelectric coefficients of random porous PZT piezoceramics with different pore volume fractions.


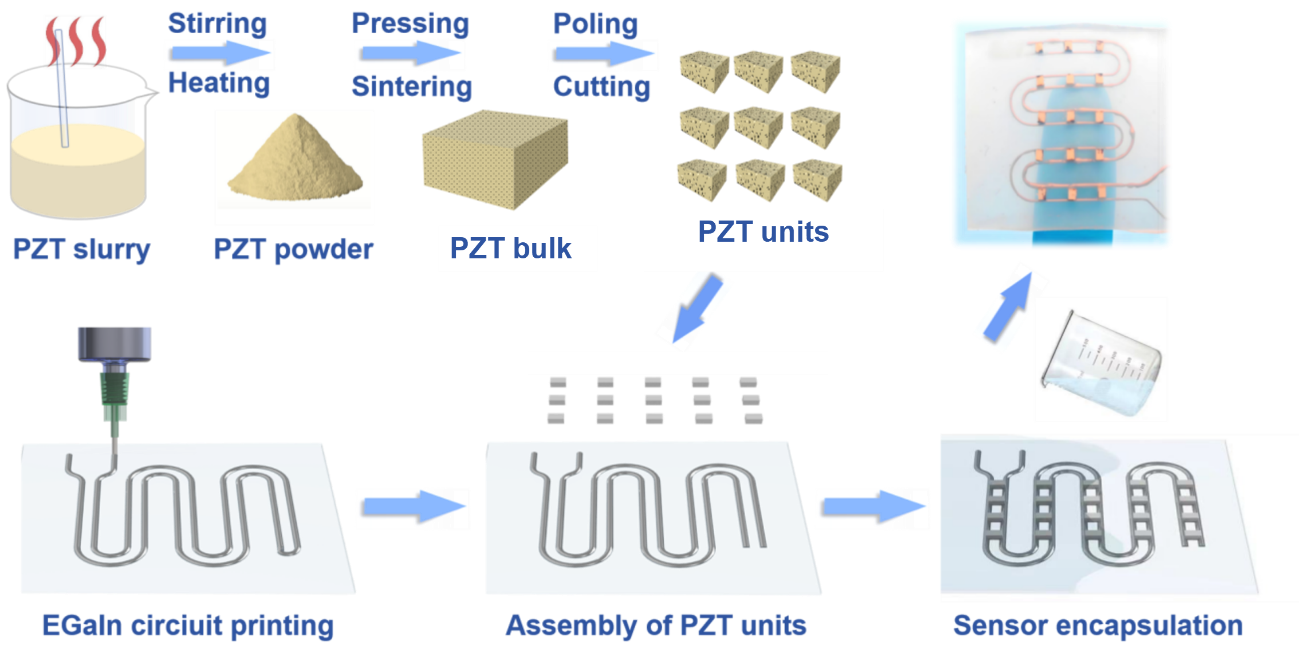


Figure S26. Schematic of Stretchable piezoelectric devices formed by PZT piezoceramic units and customizable EGaIn printed circuits.


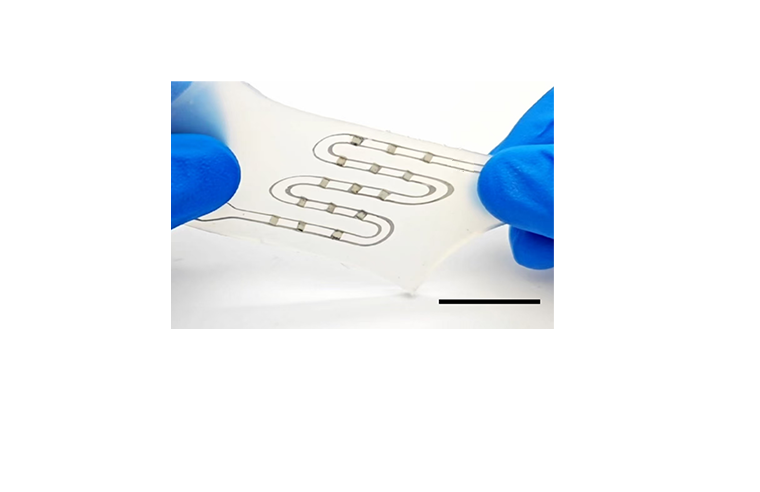


Figure S27. Optical image of the 3 × 5 array stretchable sensors subject to tensile strain. Scale bar, 3 cm.


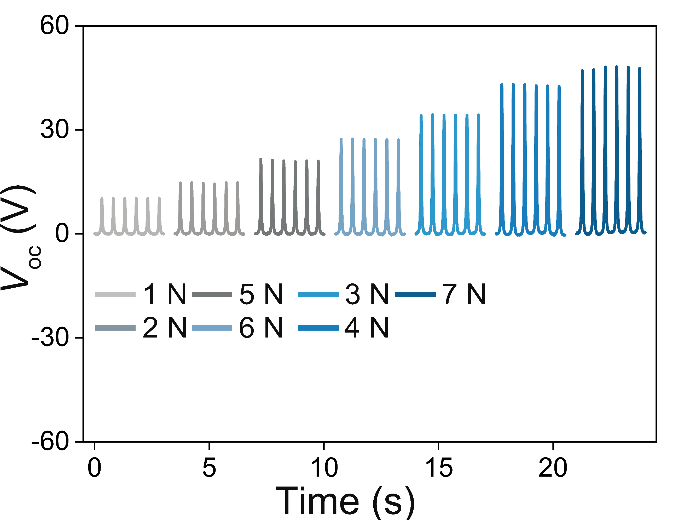


**Figure S28.** Open-circuit voltage (*V*_oc_) in forward connection at 2 Hz for a range of forces during compressive deformation.


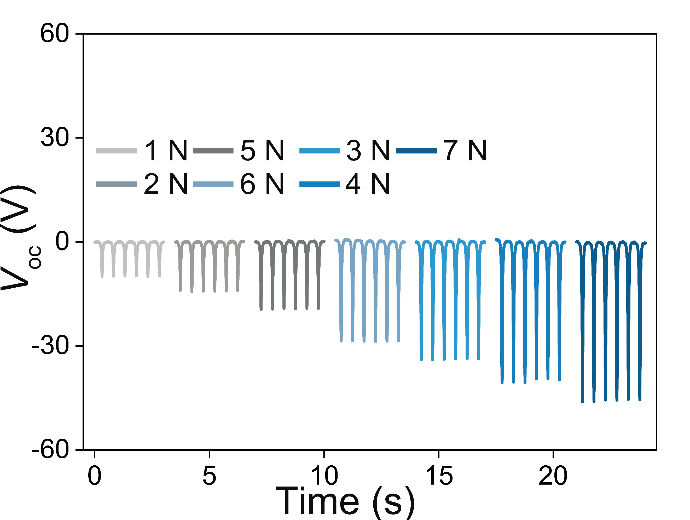


**Figure S29.** *V*_oc_ in reverse connection at 2 Hz for a range of force during compressive deformation.


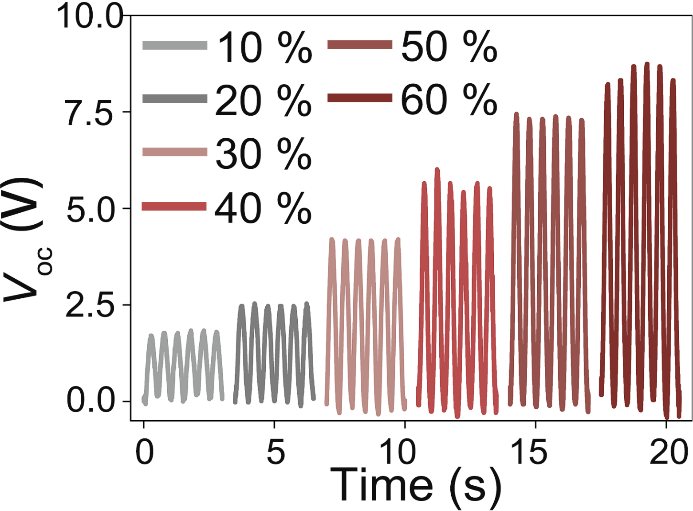


Figure S30. Variation of *V*_oc_ with strain for the 3 × 5 array stretchable piezoelectric sensor subject to tensile deformation at 2 Hz.


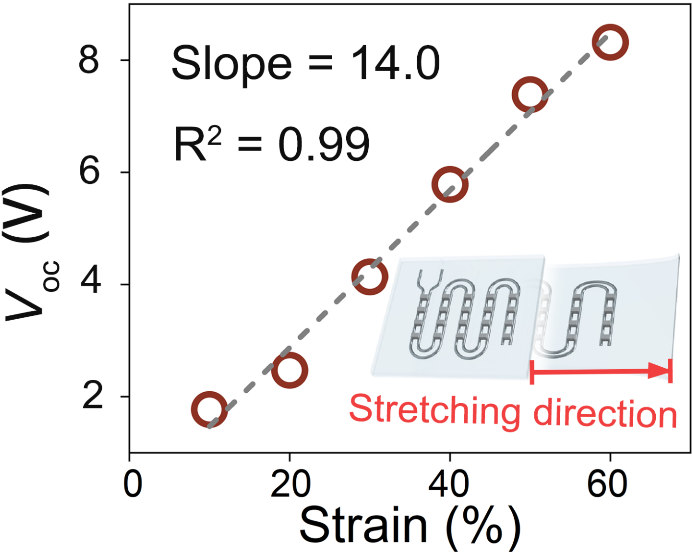


Figure S31. Linear increase in *V*oc with strain from 10% to 60% (inset: schematic of stretchable sensor subjected to tensile deformation).


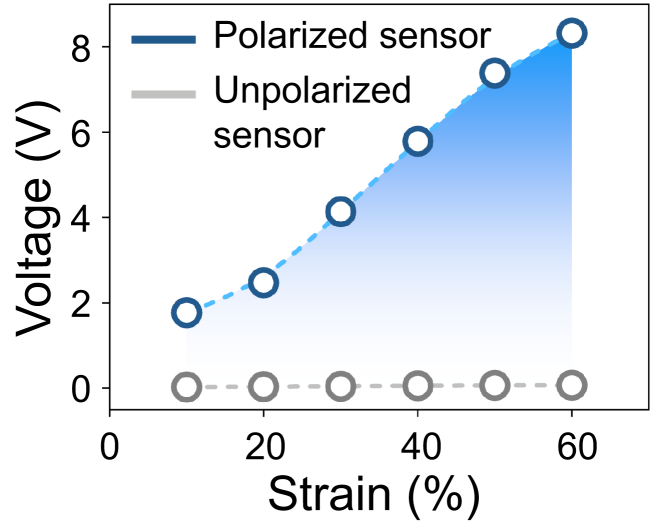


Figure S32. Comparison of open-circuit voltage, *V*_oc_, between polarized and unpolarized sensors when subjected to tensile deformation.


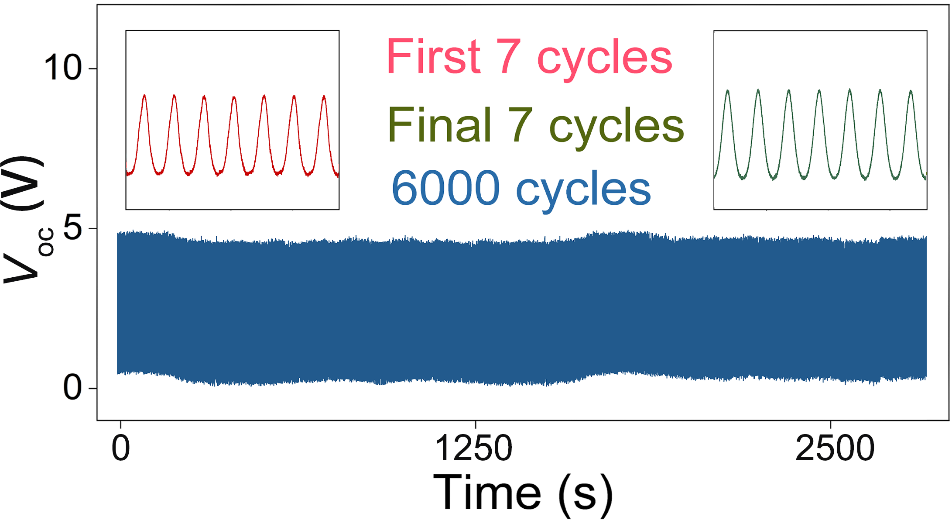


**Figure S33**. Over 6,000 stretching cycles at 40% strain and 40% relative humidity.


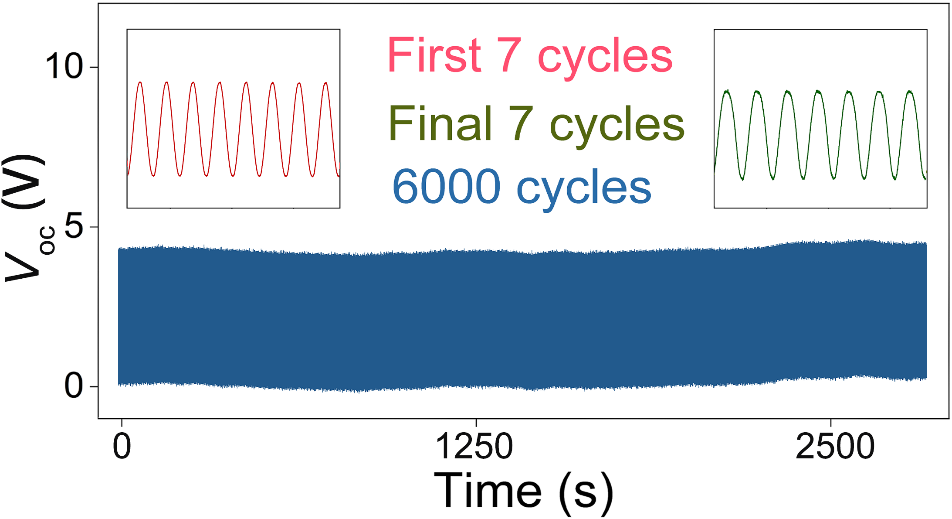


**Figure S34**. Over 6,000 stretching cycles at 40% strain and 60% relative humidity.


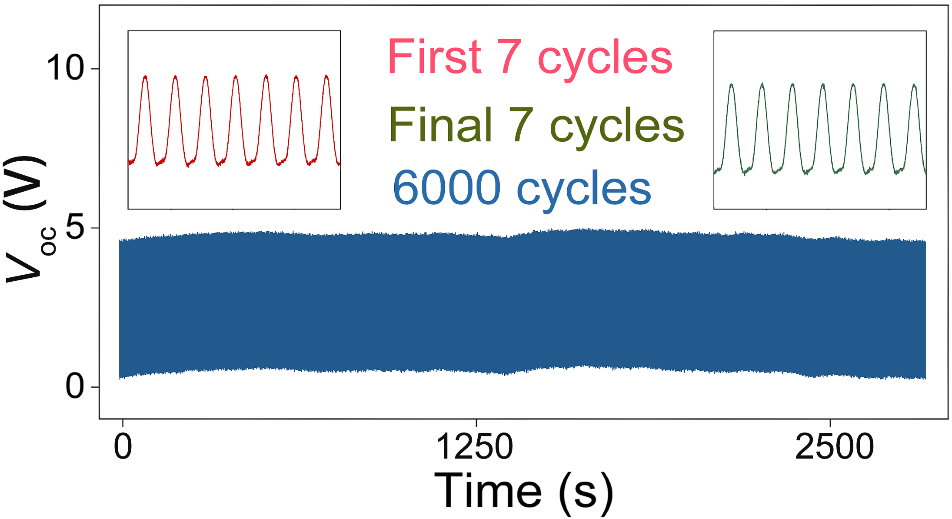


**Figure S35**. Over 6,000 stretching cycles at 40% strain and 80% relative humidity.


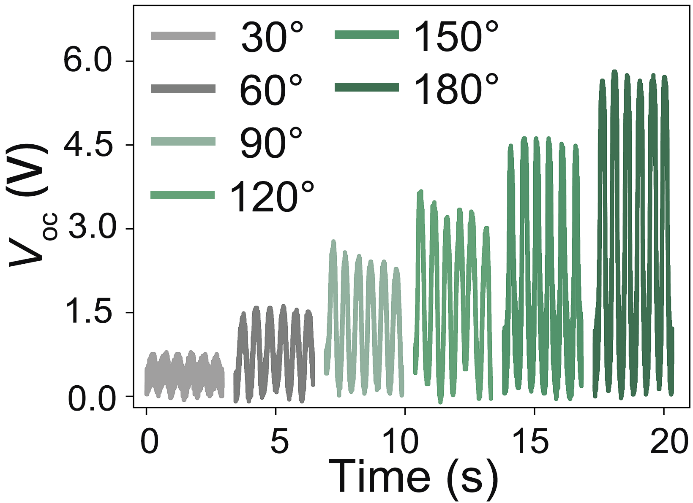


Figure S36. Variation of *V*_oc_ with strain for the 3 × 5 array stretchable piezoelectric sensor subject to torsional deformation at 2 Hz.


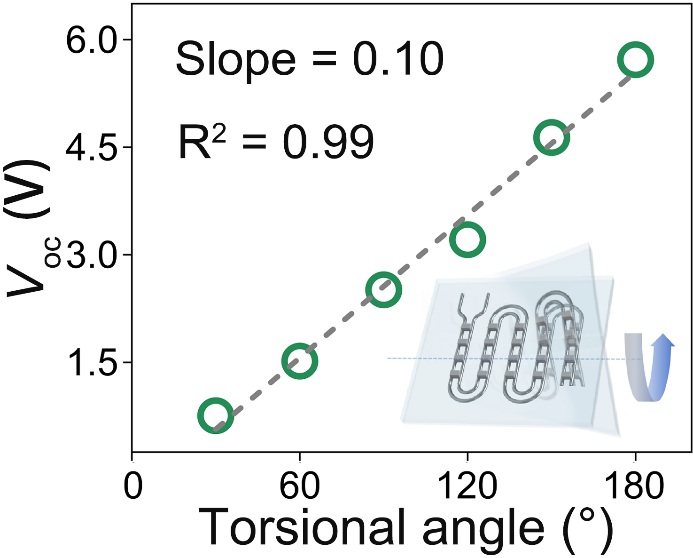


Figure S37. Linear increase in *V*oc with angle from 0° to 180° (inset: schematic of stretchable sensor subjected to tensile deformation).


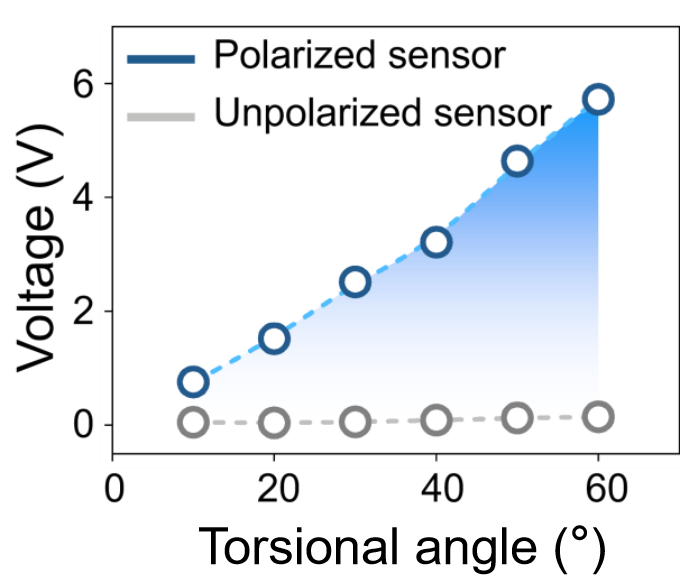


Figure S38. Comparison of *V*_oc_ between polarized and unpolarized sensors when subjected to torsional deformation.


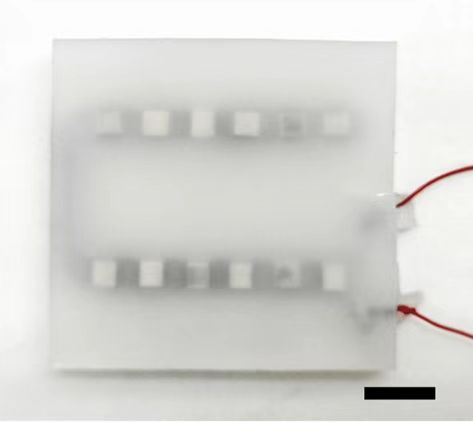


Figure S39. Optical image of the optimized sensor by topology optimization during tensile deformation. Scale bar, 6 mm.


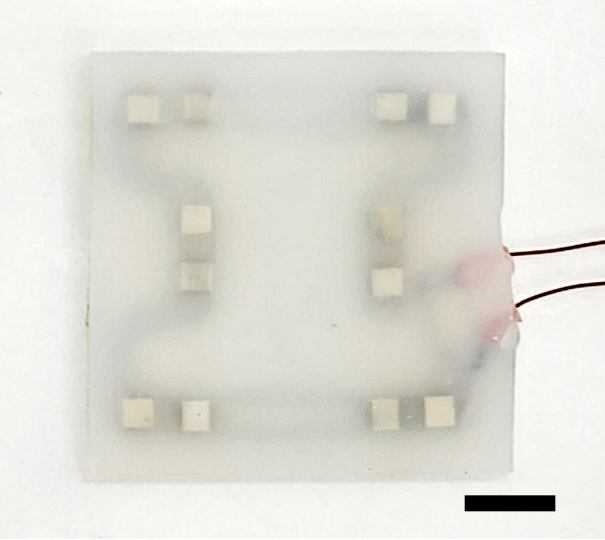


**Figure S40.** Optical image of the optimized sensor by topology optimization during torsional deformation. Scale bar, 6 mm.


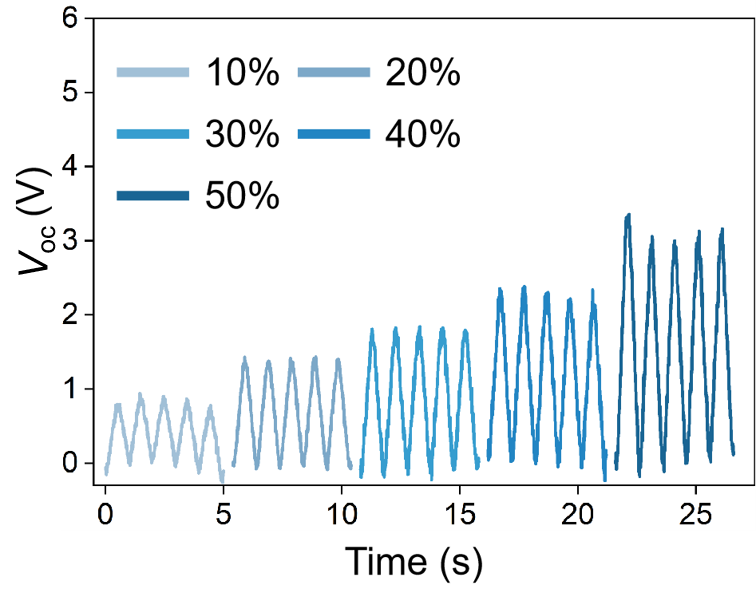


Figure S41. *V*_oc_ of the 1st topological iteration optimized sensor at a strain of 10%, 20%, 30%, 40%, 50%, and at a frequency of 1 Hz.


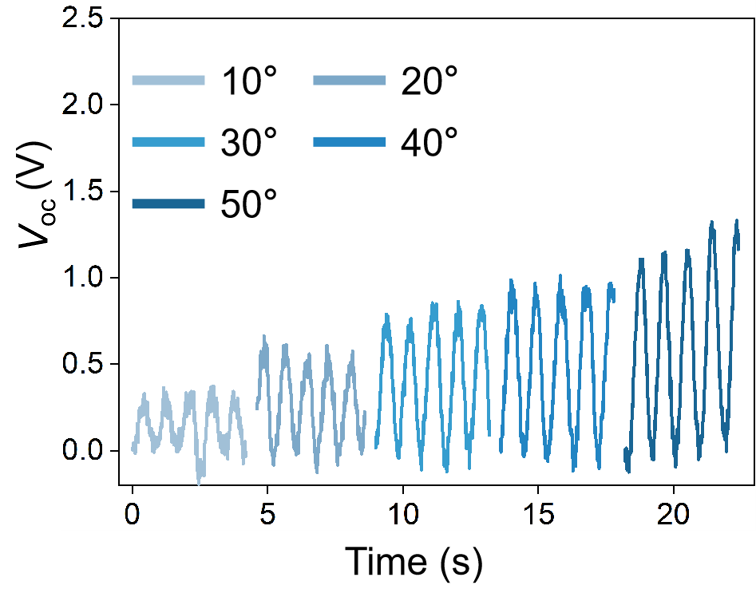


Figure S42. *V*_oc_ of the 1st topological iteration optimized sensor at a strain of 10°, 20°, 30°, 40°, 50°, and at a frequency of 1 Hz.

Table S1. Calculated tangential component of shear force (N m^-2^) generated by the relative motion of the EGaIn and the nozzle.

| **Flow rate (mL h^-1^)** | **Printing speed (m min^-1^)** | | | | |
| --- | --- | --- | --- | --- | --- |
|  | **0.4** | **0.8** | **1.2** | **1.6** | **2.0** |
| **Height (*h*) = 0.01mm** | |  |  |  |  |
| **3** | 0.041 | 0.126 | 0.234 | 0.364 | 0.513 |
| **6** | 0.029 | 0.088 | 0.163 | 0.252 | 0.354 |
| **9** | 0.023 | 0.071 | 0.132 | 0.204 | 0.287 |
| **12** | 0.020 | 0.062 | 0.114 | 0.176 | 0.247 |
| **15** | 0.018 | 0.055 | 0.101 | 0.157 | 0.220 |
| **Height (*h*) = 0.02mm** | |  |  |  |  |
| **3** | 0.040 | 0.115 | 0.216 | 0.338 | 0.479 |
| **6** | 0.028 | 0.079 | 0.148 | 0.230 | 0.325 |
| **9** | 0.022 | 0.064 | 0.119 | 0.185 | 0.261 |
| **12** | 0.019 | 0.055 | 0.102 | 0.159 | 0.224 |
| **15** | 0.017 | 0.049 | 0.091 | 0.141 | 0.199 |
| **Height (*h*) = 0.03mm** | |  |  |  |  |
| **3** | 0.040 | 0.117 | 0.222 | 0.351 | 0.502 |
| **6** | 0.027 | 0.080 | 0.150 | 0.234 | 0.333 |
| **9** | 0.022 | 0.064 | 0.120 | 0.187 | 0.264 |
| **12** | 0.019 | 0.055 | 0.102 | 0.159 | 0.225 |
| **15** | 0.017 | 0.049 | 0.091 | 0.141 | 0.199 |

Table S2. Calculated value of the tangential component of shear force (N m^-2^) between the EGaIn and the substrate.

| **Height (mm)** | **Printing speed (m min^-1^)** | | | | |
| --- | --- | --- | --- | --- | --- |
|  | **0.4** | **0.8** | **1.2** | **1.6** | **2.0** |
| **0.01** | 1.21 | 2.41 | 3.62 | 4.83 | 6.04 |
| **0.02** | 0.60 | 1.21 | 1.81 | 2.41 | 3.02 |
| **0.03** | 0.40 | 0.80 | 1.21 | 1.61 | 2.01 |

Table S3. Predicted value of calculated trace width (*w*, mm).

| **Flow rate (mL h^-1^)** | **Printing speed (m min^-1^)** | | | | |
| --- | --- | --- | --- | --- | --- |
|  | **0.4** | **0.8** | **1.2** | **1.6** | **2.0** |
| **Height (*h*) = 0.01mm** | |  |  |  |  |
| **3** | 0.494 | 0.328 | 0.268 | 0.232 | - |
| **6** | 0.698 | 0.463 | 0.378 | 0.328 | 0.293 |
| **9** | 0.855 | 0.568 | 0.463 | 0.401 | 0.359 |
| **12** | 0.988 | 0.655 | 0.535 | 0.463 | 0.414 |
| **15** | 1.104 | 0.733 | 0.598 | 0.518 | 0.463 |
| **Height (*h*) = 0.02mm** | |  |  |  |  |
| **3** | 0.463 | 0.328 | 0.268 | 0.232 | 0.207 |
| **6** | 0.655 | 0.463 | 0.378 | 0.328 | 0.293 |
| **9** | 0.803 | 0.568 | 0.463 | 0.401 | 0.359 |
| **12** | 0.927 | 0.655 | 0.535 | 0.463 | 0.414 |
| **15** | 1.036 | 0.733 | 0.598 | 0.518 | 0.463 |
| **Height (*h*) = 0.03mm** | |  |  |  |  |
| **3** | - | 0.320 | 0.261 | 0.226 | - |
| **6** | - | 0.452 | 0.369 | 0.320 | 0.286 |
| **9** | - | 0.554 | 0.452 | 0.392 | 0.350 |
| **12** | - | 0.640 | 0.522 | 0.452 | 0.405 |
| **15** | - | 0.715 | 0.584 | 0.506 | 0.452 |

**Table S4.** Experimental value of measured *w* (mm).

| **Flow rate** **(mL h^-1^)** | **Printing speed** **(m min^-1^)** | | | | |
| --- | --- | --- | --- | --- | --- |
|  | **0.4** | **0.8** | **1.2** | **1.6** | **2.0** |
| **Height (*h*) = 0.01mm** | |  |  |  |  |
| **3** | 0.427 | 0.303 | 0.253 | 0.214 | - |
| **6** | 0.612 | 0.409 | 0.336 | 0.295 | 0.265 |
| **9** | 0.740 | 0.524 | 0.417 | 0.366 | 0.324 |
| **12** | - | 0.567 | 0.467 | 0.418 | 0.391 |
| **15** | - | 0.625 | 0.545 | 0.467 | 0.424 |
| **Height (*h*) = 0.02mm** | |  |  |  |  |
| **3** | 0.408 | 0.332 | 0.273 | 0.233 | 0.194 |
| **6** | 0.574 | 0.419 | 0.350 | 0.306 | 0.282 |
| **9** | 0.689 | 0.532 | 0.414 | 0.378 | 0.357 |
| **12** | - | 0.575 | 0.483 | 0.430 | 0.415 |
| **15** | - | 0.649 | 0.556 | 0.472 | 0.430 |
| **Height (*h*) = 0.03mm** | |  |  |  |  |
| **3** | - | 0.342 | 0.273 | 0.231 | - |
| **6** | - | 0.446 | 0.363 | 0.332 | 0.297 |
| **9** | - | 0.542 | 0.435 | 0.375 | 0.353 |
| **12** | - | 0.574 | 0.469 | 0.445 | 0.419 |
| **15** | - | 0.618 | 0.586 | 0.465 | 0.443 |

**Table S5.** Relative error, *ε*, of *w* between the prediction results and the experimental result.

| **Flow rate (mL h^-1^)** | **Printing speed (m min^-1^)** | | | | |
| --- | --- | --- | --- | --- | --- |
|  | **0.4** | **0.8** | **1.2** | **1.6** | **2.0** |
| **Height (*h*) = 0.01mm** | |  |  |  |  |
| **3** | 13.53% | 7.49% | 5.26% | 7.51% | - |
| **6** | 12.38% | 11.78% | 11.23% | 9.94% | 9.55% |
| **9** | 13.48% | 7.70% | 10.10% | 8.78% | 9.82% |
| **12** | - | 13.43% | 12.73% | 9.72% | 5.60% |
| **15** | - | 14.68% | 8.85% | 9.90% | 8.54% |
| **Height (*h*) = 0.02mm** | |  |  |  |  |
| **3** | 11.93% | -1.41% | -1.90% | -0.76% | 6.38% |
| **6** | 12.42% | 9.60% | 7.60% | 6.64% | 3.73% |
| **9** | 14.21% | 6.28% | 10.60% | 5.82% | 0.67% |
| **12** | - | 12.24% | 9.75% | 7.14% | -0.18% |
| **15** | - | 11.48% | 6.98% | 8.82% | 7.16% |
| **Height (*h*) = 0.03mm** | |  |  |  |  |
| **3** | - | -7.01% | -4.38% | -1.96% | - |
| **6** | - | 1.35% | 1.71% | -3.75% | -3.76% |
| **9** | - | 2.23% | 3.73% | 4.17% | -0.72% |
| **12** | - | 10.31% | 10.19% | 1.67% | -3.46% |
| **15** | - | 13.56% | -0.28% | 8.08% | 2.15% |

**Table S6.** Comparison of wettability on different surfaces.

|  | **Ecoflex** | **PDMS** | **Glass** | **Paper** |
| --- | --- | --- | --- | --- |
| **Advancing contact angle, *θ*_adv_** | 156.2° | 146.3° | 155.8° | 151.4° |
| **Receding contact angle, *θ*_rec_** | 150.2° | 140.4° | 149.1° | 146.2° |

Table S7. Calculated value of the tangential component of shear force (N m^-2^) between the EGaIn and the PDMS.

| **Height (mm)** | **Printing speed (m min^-1^)** | | | | |
| --- | --- | --- | --- | --- | --- |
|  | **0.4** | **0.8** | **1.2** | **1.6** | **2.0** |
| **0.01** | 1.10 | 2.21 | 3.31 | 4.42 | 5.52 |
| **0.02** | 0.55 | 1.10 | 1.66 | 2.21 | 2.76 |
| **0.03** | 0.37 | 0.74 | 1.10 | 1.47 | 1.84 |

Table S8. Calculated value of the tangential component of shear force (N m^-2^) between the EGaIn and the glass.

| **Height (mm)** | **Printing speed (m min^-1^)** | | | | |
| --- | --- | --- | --- | --- | --- |
|  | **0.4** | **0.8** | **1.2** | **1.6** | **2.0** |
| **0.01** | 1.21 | 2.42 | 3.63 | 4.84 | 6.05 |
| **0.02** | 0.60 | 1.21 | 1.81 | 2.42 | 3.02 |
| **0.03** | 0.40 | 0.81 | 1.21 | 1.61 | 2.07 |

Table S9. Calculated value of the tangential component of shear force (N m^-2^) between the EGaIn and the paper.

| **Height (mm)** | **Printing speed (m min^-1^)** | | | | |
| --- | --- | --- | --- | --- | --- |
|  | **0.4** | **0.8** | **1.2** | **1.6** | **2.0** |
| **0.01** | 1.16 | 2.33 | 3.49 | 4.66 | 5.82 |
| **0.02** | 0.58 | 1.16 | 1.75 | 2.33 | 2.91 |
| **0.03** | 0.39 | 0.78 | 1.16 | 1.55 | 1.94 |

**Table S10.** Comparison of maximum stretchablity, thinnest trace width, and robustness of stretchable circuits with previously reported work.

| **Material** | **Maximum stretchablity** | **Thinnest trace width (μm)** | **Mechanical test** |
| --- | --- | --- | --- |
| This work | 200% | 40.2 | 22,000 cycles at 40% strain |
| Ga–In^[1]^ | 1000% | - | 1,500 cycles at 100% strain |
| PU/Ag^[2]^ | 350% | 1483 | 1000 cycles at 100% strain |
| AgNWs^[3]^ | 114% | 1300 | 8,000 cycles at 10% strain |

**Table S11.** Comparison of *V*_oc_ and durability of stretchable piezoelectric sensors with previously reported work.

| **Material** | ***V*_oc_ in compression (V)** | **Mechanical test** |
| --- | --- | --- |
| This work | 47.4 (compression), 9.0 (tension) | 5,000 compression cycles, 13,000 tension cycles |
| [Me_3_NCH_2_Cl]CdCl_3_ (TMCM-CdCl₃)^[4]^ | 103.0 (compression) | 10,000 compression cycles |
| PVDF^[5]^ | 4.0 (compression) | 5,000 compression cycles |
| PVDF/DA^[6]^ | 85.1 (compression) | 10,000 compression cycles |
| PVDF^[7]^ | 1.0 (tension) | 20,000 tension cycles |
| BTO-PU^[8]^ | 9.3 (tension) | 9,000 tension cycles |
| PDMS-CNT/P(VDF-TrFE)^[9]^ | 1.4 (tension) | 4,000 tension cycles |

**Table S12.** Physics, boundary condition, mesh and solver settings used in COMSOL modelling.

| **Item** | **Setting** | |
| --- | --- | --- |
| Software | COMSOL Multiphysics 6.0 | |
| Physics interfaces | Solid Mechanics, Electrostatics, Multiphysics - Piezoelectric Effect | |
| Mechanical boundary condition | Fixed constraint | |
| Loading method | Tensile deformation | Torsional deformation |
| Loading magnitude | 13 mm | 50° |
| Electrical boundary condition | Charge conservation; Zero charge; Ground | |
| Multiphysics | Electromechanical coupling (piezoelectric effect) | |
| Mesh | Normal | |
| Solver | Stationary solver | |
| Study type | Stationary | |

**Table S13.** Materials parameters of Ecoflex used in COMSOL modelling.

| **Parameter** | **Value** | **Unit** |
| --- | --- | --- |
| Relative permittivity | 2.75 | - |
| Coefficient of thermal expansion | 9 × 10^-4^ | 1/K |
| Density | 1040 | kg/m^3^ |
| Young’s modulus | 750 | Pa |
| Poisson’s ratio | 0.49 | - |

**Table S14.** Materials parameters of PZT used in COMSOL modelling.

| **Parameter** | **Value** | **Unit** |
| --- | --- | --- |
| Elastic stiffness matrix (Voigt notation) | {120.3, 75.2, 120.3, 75.1, 75.1, 110.9, 0, 0, 0, 21.1, 0, 0, 0, 0, 21.1, 0, 0, 0, 0, 0, 22.6} | GPa |
| Piezoelectric coupling matrix (Voigt notation) | {0, 0, -5.4, 0, 0, -5.4, 0, 0, 15.8, 0, 12.3, 0, 12.3, 0, 0, 0, 0, 0} | C/m^2^ |
| Relative permittivity | 1460 | - |
| Density | 7750 | kg/m^3^ |
| Poisson’s ratio | 0.34 | - |

**Table S15** Comparison of the mechanical properties of encapsulation materials.

| **Material** | **Young’s Modulus （MPa）** | **Elongation at Break** | **Density（kg/m^3^）** | **Poisson’s ratio** |
| --- | --- | --- | --- | --- |
| Ecoflex 00-30^[10]^ | ~ 0.1 | ~ 900% | 1040 | 0.49 |
| PDMS^[11]^ | ~ 0.4 | ~ 70% | 970 | 0.49 |
| TPU^[12]^ | ~ 5 | ~ 700% | 1100 | ~ 0.45 |

References

[1] S. Liu, D. S. Shah, R. Kramer-Bottiglio, *Nature Materials* **2021**, 20, 851.

[2] J. Lv, G. Thangavel, Y. Xin, D. Gao, W. C. Poh, S. Chen, P. S. Lee, *Nature Communications* **2023**, 14, 7132.

[3] X. He, N. Zhou, Y. Li, P. Xiong, S. Zhang, Z. Ma, *Materials Advances* **2023**, 4, 1978.

[4] J.-Q. Luo, H.-F. Lu, Y.-J. Nie, Y.-H. Zhou, C.-F. Wang, Z.-X. Zhang, D.-W. Fu, Y. Zhang, *Nature Communications* **2024**

[5] Y. Zhang, C. Liu, B. Jia, D. Ma, X. Tian, Y. Cui, Y. Deng, *npj Flexible Electronics* **2024**, 8, 23.

[6] J. Xiong, L. Wang, F. Liang, M. Li, Y. Yabuta, M. A. Iqbal, G. Mayakrishnan, J. Shi, I. S. Kim, *Advanced Fiber Materials* **2024**, 6, 1212.

[7] W. Fan, R. Lei, H. Dou, Z. Wu, L. Lu, S. Wang, X. Liu, W. Chen, M. Rezakazemi, T. M. Aminabhavi, Y. Li, S. Ge, *Nature Communications* **2024**, 15, 3509.

[8] S. Siddiqui, H. B. Lee, D. I. Kim, L. T. Duy, A. Hanif, N. E. Lee, *Adv. Energy Mater.* **2017**, 8, 1701520.

[9] J. H. Lee, K. Y. Lee, M. K. Gupta, T. Y. Kim, D. Y. Lee, J. Oh, C. Ryu, W. J. Yoo, C. Y. Kang, S. J. Yoon, J. B. Yoo, S. W. Kim, *Adv. Mater.* **2014**, 26, 765.

[10] Smooth-On Inc., Ecoflex™ 00-30 Platinum Silicone Rubber: Technical Data Sheet, Smooth-On, USA

[11] I. D. Johnston, D. K. McCluskey, et al., J. Micromech. Microeng. **2014**, 24, 035017

[12] C. Emminger, U. D. Çakmak, R. Preuer, I. Graz, Z. Major, Materials **2021**, 14, 763
